# The Novel IGF-1R Inhibitor PB-020 Acts Synergistically with Anti-PD-1 and Mebendazole against Colorectal Cancer

**DOI:** 10.3390/cancers14235747

**Published:** 2022-11-23

**Authors:** Bo Kang, Xiaobing Zhang, Weibing Wang, Shiqi She, Wenjie Chen, Cheng Chen, Yisha Wang, Xiaoyun Pan, Ouyuan Xu, Yingjie Wang

**Affiliations:** 1 State Key Laboratory for Diagnosis and Treatment of Infectious Diseases, National Clinical Research Center for Infectious Diseases, National Medical Center for Infectious Diseases, Collaborative Innovation Center for Diagnosis and Treatment of Infectious Diseases, The First Affiliated Hospital, School of Medicine, Zhejiang University, Hangzhou 310003, China; 2 Center for Integrated Oncology and Precision Medicine, Affiliated Hangzhou First People’s Hospital, Zhejiang University School of Medicine, Hangzhou 310006, China; 3 Department of Colorectal and Anal Surgery, The First Affiliated Hospital, School of Medicine, Zhejiang University, Hangzhou 310003, China; 4 Zhejiang Museum of Natural History, Hangzhou 310014, China; 5 College of Animal Sciences, Zhejiang University, Hangzhou 310058, China; 6 Cancer Center, Zhejiang University, Hangzhou 310058, China

**Keywords:** colorectal cancer, IGF-1R, PB-020, MBZ, PD-1, combination therapy, PI3K/AKT

## Abstract

**Simple Summary:**

Colorectal cancer (CRC) is generally diagnosed at an advanced stage, when chemo- and targeted therapies can only achieve a limited increase in overall survival for these patients. The activation of insulin/IGF-dependent pathways has been established as a key step that contributes to several mechanisms of CRC resistance to conventional and targeted therapeutic drugs. In this study, we tested the combinatory effect of the novel IGF-1R inhibitor PB-020 and MBZ or anti-PD-1 against CRC, respectively. The PB-020/MBZ combination significantly reduced the pAKT1-S473 level, dampened the viability of CRC cells and blocked CRC progression in a xenograft model. More importantly, the PB-020/anti-PD-1 combination synergistically blocked CRC propagation in the MC38 murine colon carcinoma model. RNA-seq analysis indicated that PB-020 and the anti-PD-1 antibody synergistically suppressed the transcription of the PI3K/AKT pathway genes. These findings establish a preclinical proof-of-concept for tackling CRC using a combination of PB-020 and clinically approved anticancer drugs.

**Abstract:**

CRC is one of the leading causes of cancer mortality worldwide. Chemotherapy is widely used for the treatment of CRC, but its efficacy remains unsatisfactory, mainly due to drug resistance. Therefore, it is urgent to develop new strategies to overcome drug resistance. Combination therapy that aims to achieve additive or synergistic therapeutic effects is an effective approach to tackle the development of drug resistance. Given its established roles in tumor development, progression and metastasis, IGF-1R is a promising drug target for combination therapy against CRC. In this study, we revealed that the novel IGF-1R inhibitor PB-020 can act synergistically with mebendazole (MBZ) to reduce the viability of CRC cells and block xenograft CRC progression. Moreover, the PB-020/anti-PD-1 combination synergistically blocked CRC propagation in the MC38 murine colon carcinoma model. Both combination therapies potently suppressed the PI3K/AKT signaling pathway genes in CRC that may be associated with the development of drug resistance. Our findings establish a preclinical proof-of-concept for combating CRC using combined multi-target treatment with PB-020 and clinical anticancer drugs, which may provide useful clues for clinical trials to evaluate the efficacy and safety of these drug combinations in CRC patients.

## 1. Introduction

Colorectal cancer (CRC) is the third most common malignancy in humans. Chemotherapy is mainly used for the treatment of CRC. Despite significant advances in therapies against CRC over the past few decades, drug resistance is still a major hurdle for treating CRC [1]. Therefore, one of the greatest challenges is to identify new therapeutic targets and compounds that are able to restore the drug sensitivity of CRC cells. It was reported that dual targeting of human epidermal growth factor receptor 3 (HER3) and epidermal growth factor receptor (EGFR) could overcome anti-EGFR resistance in CRC tumors [2], indicating that dual targeting therapy maybe an effective approach to overcome CRC drug resistance. Since many individual targets have been reported as effective in CRC therapy, a combination of these targets could be a promising method to reverse CRC drug resistance.

The important role of the insulin-like growth factor 1 receptor (IGF-1R) in malignant tumors has been fully established. The increase in IGF-1R activity promotes the proliferation, migration and invasion of cancer cells, and is related to tumor metastasis, drug resistance, poor prognosis and shortened survival time in patients with multiple cancers [3]. In particular, IGF-1R was considered as a promising drug target for CRC therapy [4], and the specific IGF-1R inhibitor picropodophyllin (PPP, or AXL1717) strongly inhibited the proliferation and migration of multiple CRC cell lines in a dose-dependent manner [5]. In a recent work, we designed a novel orally available IGF-1R inhibitor, PB-020, which shares identical pharmacophores and target binding sites with PPP but exhibits better pharmacokinetic properties and blood–brain barrier (BBB) penetration capabilities than PPP by replacing a few hydrogen atoms with deuterium and fluorine atoms [6]. PB-020 preferentially inhibited the proliferation of cancer stem cell-like oligodendrocyte precursor cells (OPCs) over their non-OPC counterparts [6], indicating its potential in targeting cancer stem cells. These observations together indicate that PB-020 is a good candidate for combination therapy against CRC.

Mebendazole (MBZ) is a benzimidazole derivative approved by the Food and Drug Administration (FDA). It has an excellent safety profile and has been widely used in the treatment of parasitic infections. The anthelmintic effect of MBZ is mainly attributed to its ability to bind to tubulin and inhibit microtubule polymerization, which is also related to its anti-proliferative activity against cancer cells, including CRC cells [7]. Several in vitro studies have shown that MBZ inhibits a variety of factors related to tumor progression, such as tubulin polymerization, matrix metalloproteinases, angiogenesis, pro-survival pathways, and multi-drug resistance protein transporters [8]. MBZ not only exhibits direct cytotoxic activity, but also can cooperate with ionizing radiation and different chemotherapeutic drugs to stimulate anti-tumor immune responses. In vivo, MBZ alone or in combination with chemotherapy can decrease or completely prevent tumor growth, significantly reduce metastasis and invasion, and thereby improve survival [9]. Fenbendazole (FBZ), an MBZ analogue that is used to treat pinworm infection, also exhibits anticancer effects in cancer cell lines (including CRC [10]), animal tumor models, and clinical trials [8].

Recently, anti-tumor immunity emerged as a very promising therapeutic target in combination with chemotherapy or targeted therapy [11,12]. Programmed death-1 (PD-1; CD279) is an inhibitory receptor induced in activated T cells. PD-1 participates in peripheral tolerance through its ligands PD-L1 and PD-L2, but also impairs anti-tumor immunity. Blocking antibodies against PD-1 or its ligands have revolutionized cancer immunotherapy [13]. Several clinical trials have been conducted to treat CRC with anti-PD-1 monoclonal antibodies [14]. Recently, a combination therapy that consists of anti-PD-1 antibody and a multiple receptor tyrosine kinase inhibitor (Foretinib) was shown to significantly inhibit CRC tumor growth and relapse in mice, and to prolong overall survival [15].

In this study, we tested the combinatory effect of the IGF-1R inhibitor PB-020 and MBZ or anti-PD-1 against CRC, respectively.

## 2. Materials and Methods

### 2.1. Cell Lines and Reagents

Human colorectal adenocarcinoma HT-29, WE480, HCT 116, LoVo and SW620 cell lines and mouse colorectal adenocarcinoma MC38 cells were purchased from the National Collection of Authenticated Cell Cultures (https://cellbank.org.cn/, accessed on 20 August 2022), and authenticated by STR before the sale. Cells were maintained in a 37 °C incubator with 5% CO_2_. The cell culture media were as follows: McCoy’s 5A (GIBCO, 16600082, Waltham, USA) with 10% FBS (Gibco, 10099-141, Waltham, USA) for HT-29 and HCT 116, L-15 (Hyclone, SH30525.01, Logan, USA) with 10% FBS (Gibco, 10099-141, Waltham, USA) for SW480 and SW620, Ham’s F-12K (Hyclone, SH30526.01) with 10% FBS (Gibco, 10099-141, Waltham, USA) for LoVo and DMEM (Hyclone, SH30243.01B) with 10% FBS (Gibco, 10099-141, Waltham, USA) for MC38, respectively. Penicillin-Streptomycin (Gibco, C14-15070-063, Waltham, USA) and plasmocin (Invitrogen, ant-mpp, Carlsbad, USA) were added to the cell culture media. All cells were checked for mycoplasma DNA every 3 months.

### 2.2. Cell Viability Assay and Combined Drug Effects

Exponentially growing cells were treated with PB-020 and/or other drugs (MBZ, FBZ and Taxol) for 3 days. Cell Counting Kit-8 (Beyotime #C0039, Shanghai, China) assays were used to test cell viability. Determination of the half inhibitory concentration (IC50) of compounds was conducted by Prism 9 software (GraphPad, San Diego, USA). Drug combination synergy was quantified using two different reference models, the Bliss independence analysis model [16] and highest single agent (HSA) model [17]. Calculations were performed using the SynergyFinder 3.0 free web-application [18] (https://synergyfinder.fimm.fi/, accessed on 20 August 2022). A Bliss value of <0 indicates antagonism, while a Bliss value of >0 indicates synergy. An HSA value of <0 indicates antagonism, while an HSA value of >0 indicates synergy.

### 2.3. Western Blotting (WB)

The cell lysates from compound-treated cancer cells were boiled for 5 min, kept on ice for 2 min, and centrifuged at 10,000 rpm for 1 min. The supernatants were separated by 10% SDS–PAGE, blotted on PVDF membranes and probed with the indicated antibodies. The signals were visualized using the Immobilon Western Chemiluminescent HRP Substrate (Millipore WBKLS0100, Danvers, USA). IGF-I Receptor β (#9750), phospho-IGF-IR β (Y1131) (#3021), AKT(Pan) (#4821), phospho-AKT (T308) (#2965), phospho-AKT (S473) (#4060), phospho-ERK1/2 (T202/Y204) (#4370), phospho-STAT3 (Y705) (#9145) were purchased from Cell Signaling Technology (Danvers, MA, USA), and GAPDH antibodies were purchased from Genscript (#A00192, Nanjing, China).

### 2.4. Mouse Xenograft Tumor Models

#### 2.4.1. HT-29 Xenograft Model

Three-week-old female BALB/c nude mice were purchased from Shanghai Experimental Animal Centre. Human colorectal adenocarcinoma HT-29 cells were injected subcutaneously at 2 × 10^6^ cells/mice, and after 7 days, when tumors had formed, the mice were divided into four groups randomly. Experimental treatments with PB-020 (25 mg/kg per day) and MBZ (10 mg/kg per day) were performed by daily administration of the compounds via oral gavage (20 μL volume of DMSO and 180 μL of corn oil), either singly or in combination until the day before sacrifice. Tumor volume was measured and calculated (1/2 × length × width × height) every three days using vernier calipers. When the measured tumor volumes in the NC group approached or exceeded 2000 mm^3^, all mice were sacrificed. No blinding was conducted throughout the experiments.

#### 2.4.2. MC38 Xenograft Model

Five-week-old female C57BL/6 mice were purchased from Shanghai Experimental Animal Centre. Mouse colorectal adenocarcinoma MC38 cells were injected subcutaneously at 2 × 10^6^ cells/mice, and after 7 days, when tumors had formed, the mice were divided into four groups randomly. Experimental treatments with PB-020 (50 mg/kg per day) were performed by daily administration of the compounds via oral gavage (20 μL volume of DMSO and 180 μL of corn oil) until the day before sacrifice, and anti-PD-1 (anti-mouse PD-1 (CD279), BE0146, BioXcell) (200 μg/mouse, at day 9, 12, 15, 18 after MC38 cell injection) was given by intraperitoneal injection. The combination treatment was a combination of the two single treatments. Tumor volume was measured and calculated (1/2 × length × width × height) every two or three days using vernier calipers. Ehen the measured tumor volumes in the NC group approached or exceeded 2000 mm^3^, all mice were sacrificed. No blinding was conducted throughout the experiments.

### 2.5. RNA Sequencing

Tumor tissues were harvested and grinded with liquid nitrogen. They were then sent to Beijing Genomics Institute (BGI) for RNA sequencing. RNA sequencing was performed by Illumina HiSeq X Ten with 150-base paired-end reads. All reads were aligned to the mouse reference genome (mm10 or hg19) using Bowtie2 [19] with the default setting. RSEM [20] was used to calculate the transcriptional expression level as fragments per kilobase per million. Genes were considered to be differentially expressed if the fold change was > 1.5 and if the two-tailed Student’s *t* test *p* was <0.05. Gene Ontology (GO) and Kyoto Encyclopedia of Genes and Genomes (KEGG) analyses [21] were implemented using the online analysis platform of BGI (https://biosys.bgi.com/, accessed on 20 August 2022).

### 2.6. Statistical Analyses

All quantitative data are shown as mean ± S.D. Two-tailed unpaired Student’s tests were used to evaluate the statistical significance of normally distributed data, and Mann–Whitney tests were used to evaluate the statistical significance of non-normally distributed data. Significances were determined as *p* < 0.05 (*), *p* < 0.01 (**), and *p* < 0.001 (***). GraphPad Prism 9.0 statistics software was used to conduct all the statistical analyses.

## 3. Results

### 3.1. PB-020 in Combination with Multiple Anticancer Drugs Potently Reduced the Viability of CRC Cells

**Figure 1:**
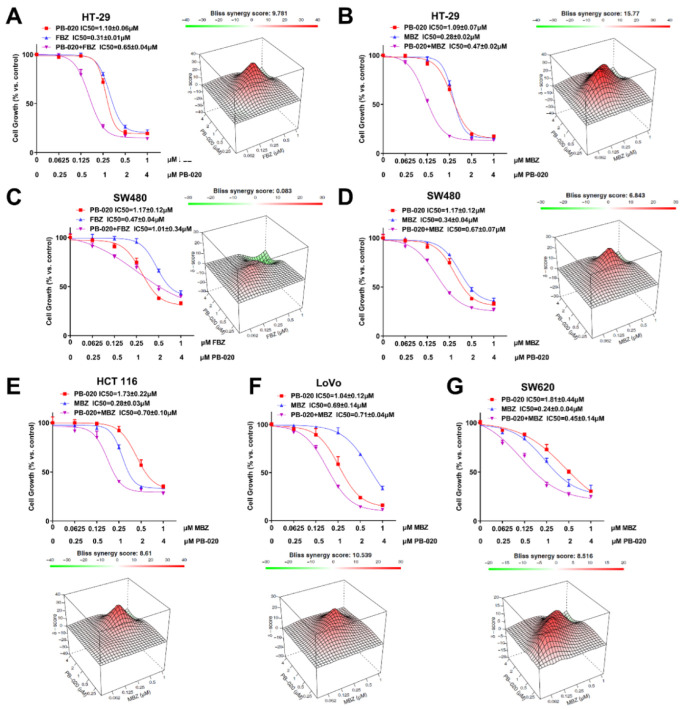
PB-020 in combination with MBZ synergistically reduced the viability of CRC cells. (**A**) HT-29 cells were treated with various concentrations (indicated under the X axis, same below) of PB-020, FBZ and their combination for 72 h, and cell viability was determined using the Cell Counting Kit-8. Bliss independence models were used to analyze the combinatorial inhibitory effect of the combinations. (**B**) HT-29 cells were treated with various concentrations of PB-020, MBZ and their combination for 72 h, and analyzed as in (**A**). (**C**) SW480 cells were treated with various concentrations of PB-020, FBZ and their combination for 72 h, and analyzed as in (**A**). (**D**) SW480 cells were treated with various concentrations of PB-020, MBZ and their combination for 72 h, and analyzed as in (**A**). (**E**) HCT 116 cells were treated with various concentrations of PB-020, MBZ and their combination for 72 h, and analyzed as in (**A**). (**F**) LoVo cells were treated with various concentrations of PB-020, MBZ and their combination for 72 h, and analyzed as in (**A**). (**G**) SW620 cells were treated with various concentrations of PB-020, Taxol and their combination for 72 h, and analyzed as in (**A**).

Three anticancer or potential anticancer compounds were selected to test their combinatory effects with PB-020 against CRC, including mebendazole (MBZ), fenbendazole (FBZ) and Taxol. The Bliss independence model (a commonly used statistical model to evaluate drug combination efficacy) was used to evaluate the combination efficacy of PB-020 and these three compounds with varying concentrations against CRC cells. The Bliss scores showed, although Taxol had an antagonistic effect with PB-020 against both HT-29 and SW480 cells (−30.928 and −9.179) (Figure S1), that MBZ and FBZ exhibited synergistic effects with PB-020 against several CRC cell lines. Specifically, for HT-29 cells, the Bliss scores of the PB-020/FBZ and PB-020/MBZ combinations were 9.781 and 15.77, respectively (the peak of Delta Bliss was from the drug concentrations close to IC50s) (Figure 1A,B and Figure S2). For SW480 cells, the corresponding Bliss scores were 0.083 and 6.843, respectively (the peak of Delta Bliss was from the drug concentrations close to IC50s) (Figure 1C,D and Figure S3). The non-parametric synergy model’s highest single agent (HSA) is useful to provide quantitative insights into how much the drug combination is better than either drug alone. In order to ensure the clinical translation of drug combinations and the accuracy of experimental data, HSA models were used to evaluate the combinations in Figure 1. As a result, the HSA scores in Supplemental Figures S1–S3 sufficiently supported the synergistic effect of PB-020 and MBZ. These data suggested that the combination treatment with PB-020/MBZ can potently reduce the viability of both HT-29 and SW480 cells, and the PB-020/FBZ combination can synergistically reduce the viability of HT-29 cells, but not SW480 cells. As the combination of PB-020 and MBZ had better combination efficacy, it was chosen for further studies.

Subsequently, the PB-020/MBZ combination was tested in another three CRC cell lines, HCT-116, LoVo and SW620. Clearly, the combination of PB-020 and MBZ showed synergistic therapeutic effects against HCT-116, LoVo and SW620 (Bliss scores of 8.61, 10.539 and 8.516, respectively) (Figure 1E–G, Figure S4 and Figure S5).

### 3.2. PB-020/MBZ Combination Potently Decreased the Level of pAKT1-S473

**Figure 2:**
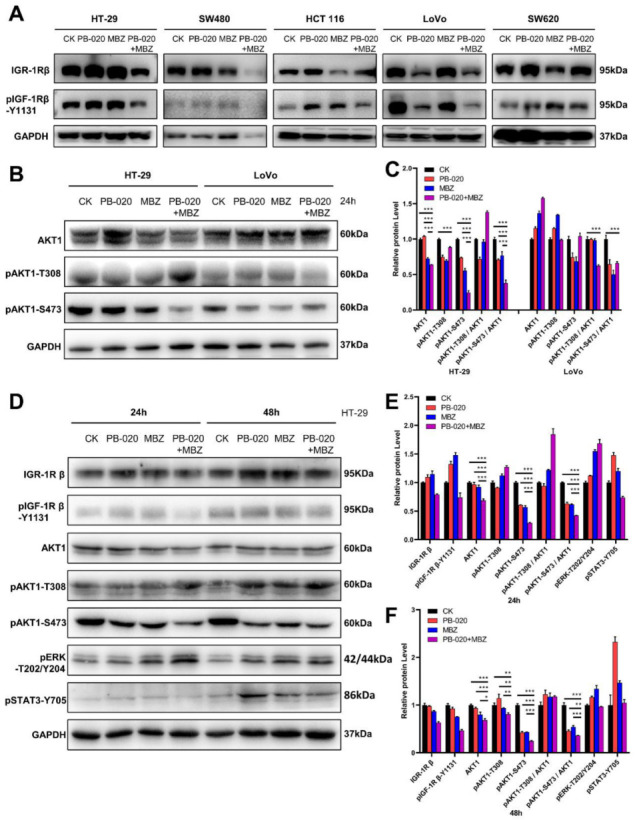
PB-020 in combination with MBZ potently reduced the level of pAKT1-S473 in CRC cells. (**A**) Five CRC cell lines were treated with a vehicle (CK), PB-020 (1 μM), MBZ (0.25 μM) or PB-020/MBZ combination for 72 h. Immunoblotting was conducted with the indicated primary antibodies. (**B**) HT-29 and LoVo cells were treated with a vehicle (CK), PB-020, MBZ or PB-020/MBZ combination for 24 h. Immunoblotting was conducted with the indicated primary antibodies. (**C**) All band intensities in (**B**) were quantified by ImageJ software and plotted as relative protein levels. (**D**) HT-29 cells were treated with a vehicle (CK), PB-020, MBZ or PB-020/MBZ combination for either 24 h or 48 h. Immunoblotting was conducted with the indicated primary antibodies. (**E**,**F**) All band intensities of 24 hour-treated samples in (**D**) were quantified by ImageJ software and plotted in (**E**), and all band intensities of 48 hour-treated samples in (**D**) were quantified by ImageJ software and plotted in (**F**). Significances were determined as *p* < 0.05 (*), *p* < 0.01 (**), and *p* < 0.001 (***).

As PB-020 is an IGF-1R inhibitor, we examined the effect of the PB-020/MBZ combination on the protein levels of IGF-1R. Interestingly, the effect appeared to vary among different CRC cell lines. Treatment with the PB-020 (1 μM) /MBZ (0.25 μM) combination for 72 h additively decreased the levels of both IGF-1R and pIGF-1R in HT-29 and LoVo cells, but not the other three CRC lines (Figure 2A and Figure S6A).

In order to gain some insights into the mechanism of action of the PB-020/MBZ combination, we conducted immunoblotting with the PB-020/MBZ combination-treated HT-29 and LoVo cell lysates to examine the status of AKT serine/threonine kinase 1 (AKT1), one of the most important downstream targets of IGF-1R. It was shown that PB-020 and MBZ potently suppressed the phosphorylation level of pAKT1-S473 in HT-29 cells, as indicated by the ratio of pAKT1-S473/AKT1 (Figure 2B,C). Further time-course study showed that compared to the 24 h treatment, treatment with the combination for 48 h amplified the potent inhibitory effect on the protein levels of IGF-1R, pIGF-1R, AKT1 and pAKT1-S473, with the pAKT1-S473 level being the most dramatically affected (Figure 2D–F and Figure S6B). In contrast, the protein levels of pERK and pSTAT3 in HT-29 cells were not reduced by the PB-020/MBZ combination.

### 3.3. Orally Administered PB-020 in Combination with MBZ Inhibited Propagation of HT-29 Cell Xenografts In Vivo

**Figure 3:**
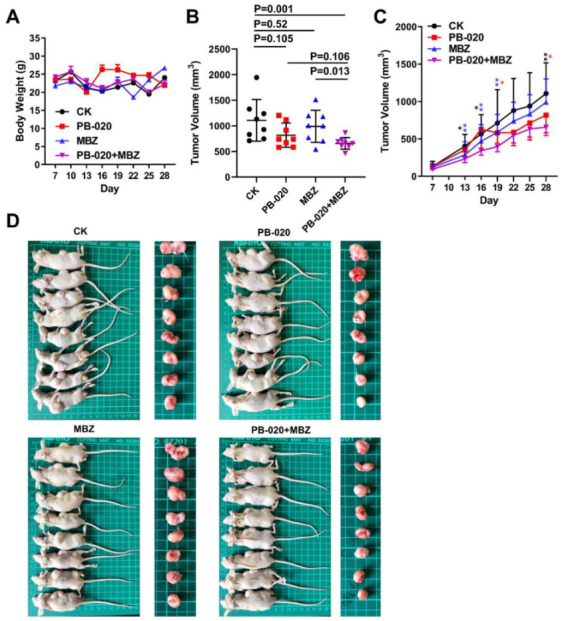
Orally administered PB-020 in combination with MBZ inhibited propagation of HT-29 cell xenografts in vivo. (**A**) Vehicle (CK), PB-020, MBZ or PB-020/MBZ combination orally administered to BALB/c nude mice, as detailed in “Materials and methods”. The effect of compound administration on body weight changes in xenograft mice is presented. (**B**) Tumor volumes at day 28 were plotted. The statistical significance of data was evaluated using the two-tailed unpaired Student’s test. (**C**) Tumor volumes as a function of time (days) were plotted. The statistical significance of data was evaluated using the two-tailed unpaired Student’s test, and differences between PB-020 + MBZ vs. CK, PB-020 + MBZ vs. PB-020 and PB-020 + MBZ vs. MBZ are shown as black, red and blue stars. Significances were determined as *p* < 0.05 (*), *p* < 0.01 (**). (**D**) Sacrificed mice (**left**) and their bearing tumors (**right**) are presented.

Next, to evaluate the effectiveness of the PB-020/MBZ combination in a xenograft animal model, we tested the effect of PB-020 and MBZ singly or in combination on the growth of HT-29 cells implanted in the nude mice. Seven days after implantation of HT-29 cells, the mice were randomly grouped and treated with a vehicle (CK), PB-020 (25 mg/kg), MBZ (10 mg/kg), or PB-020/MBZ combination by oral gavage every day for consecutive 28 days. None of these treatments exhibited discernable toxicity (Figure 3A). The antitumor effect of the combination treatment was significantly different from that of MBZ (*p* = 0.013), but not for PB-020 alone (*p* = 0.106), and neither was the effect of PB-020 (*p* = 0.105) or MBZ (*p* = 0.52) treatment alone significantly different from that of the vehicle. Nevertheless, the tumor volumes in the combination group were significantly smaller than those in the vehicle group (*p* = 0.001), indicating an additive effect between PB-020 and MBZ at the tested doses (Figure 3B–D). These data suggested that despite limited in vivo anticancer efficacy when used alone, PB-020 in combination with MBZ can potently suppress the growth of CRC in vivo.

### 3.4. PB-020 in Combination with PD-1 Blockade Inhibited Propagation of MC38 Colorectal Cancer In Vivo

Immune surveillance escaping is essential for the survival of primary tumor and circulating metastatic tumor cells. The PI3K-AKT pathway plays multiple roles in both myeloid cells of the innate immune system and lymphoid cells of the adaptive immune system. For instance, in the adaptive immune system, PI3K signaling plays a diverse and key role in T cell and B cell functions [22]. In lymphocytes, the PI3Kδ isoform, which is activated by the T cell receptor (TCR), B cell receptor (BCR), and various co-receptors and cytokine receptors, results in the activation of AKT and other effectors [23].

**Figure 4:**
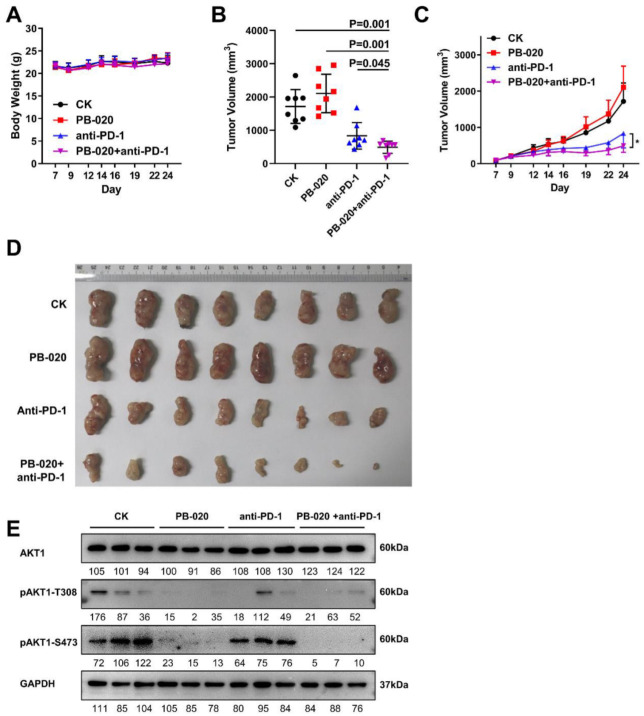
PB-020 in combination with PD-1 blockade inhibited propagation of MC38 colorectal cancer in vivo. (**A**) MC38 cells were subcutaneously implanted in C57BL/6 mice in a syngeneic mouse CRC model. The mice were administered with a vehicle (CK), PB-020, anti-PD-1 monoclonal antibody RMP1-14 or PB-020/anti-PD-1 combination, as detailed in “Materials and methods”. The effect of compound administration on body weight changes in xenograft mice is presented. (**B**) Tumor volumes at day 24 were plotted. The statistical significance of data was evaluated using the two-tailed unpaired Student’s test. (**C**) Tumor volumes as a function of time (days) were plotted. The statistical significance of data was evaluated using the two-tailed unpaired Student’s test, and differences were considered significant at * *p* < 0.05. (**D**) At the end of the experiments, the excised tumors were photographed and presented. (**E**) pAKT1 inhibitory effects of PB-020, anti-PD-1 and combined PB-020 + anti-PD-1 in MC38 tumor tissues were assessed by WB.

Given PB-020’s improved pharmacokinetic properties and AKT inhibitory efficacy, we tested the efficacy of combining PB-020 with the anti-PD-1 monoclonal antibody RMP1-14 on the growth of mouse MC38 CRC cells subcutaneously implanted in C57BL/6 mice in a syngeneic mouse CRC model. Without obvious toxicity, the combination of PB-020 with PD-1 blockade significantly reduced MC38 tumor growth compared with the effect of each treatment alone (Figure 4A,B,D). Furthermore, the tumor volume, as a function of time, continuously decreased in the PB-020/anti-PD-1 group and the reduction was more prominent than that in each treatment alone (Figure 4C and Figure S7), suggesting the durative and cumulative effect of this combination therapy. We further examined the inhibitory effect of PB-020 and anti-PD-1 on pAKT1-S473 activity in tumor tissues. WB showed that pAKT1-S473 activity was severely suppressed in tumor tissues by PB-020 and anti-PD-1 treatment (Figure 4E).

### 3.5. PB-020 in Combination with PD-1 Blockade Suppressed Transcription of PI3K/AKT Pathway Genes

**Figure 5:**
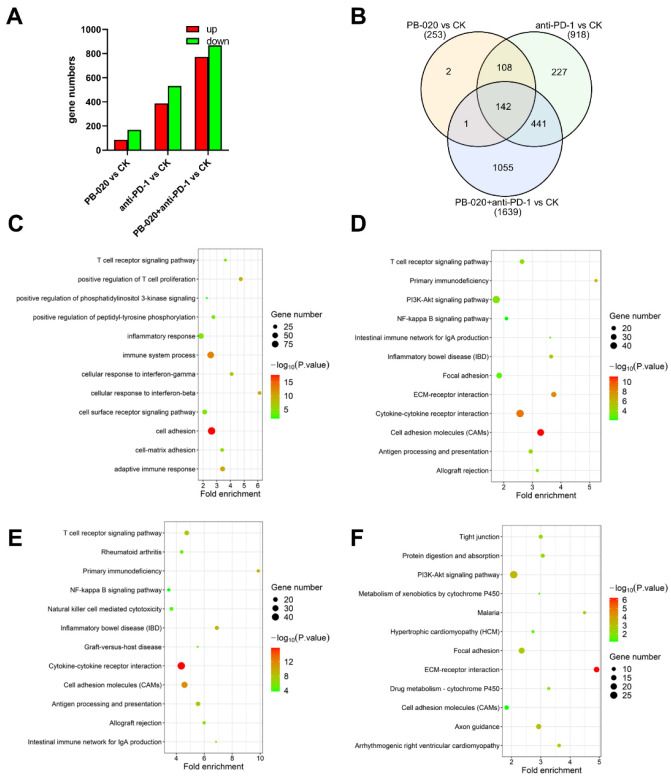
Bioinformatics analysis of DE genes in PB-020/anti-PD-1 combination versus control group mentioned in Figure 4. (**A**) Triplicate tumor tissues from the 4 groups were collected and subjected to RNA-seq analysis, and the up-regulated and down-regulated gene numbers are shown. (**B**) The Venn diagram shows the number of differentially expressed (DE) genes between the PB-020, anti-PD-1 and PB-020/anti-PD-1 combination group versus the control group, respectively. (**C**) GO biological process analysis of the identified DE genes in the PB-020/anti-PD-1 combination versus control group. (**D**) Enrichment analysis of the KEGG pathway of the identified DE genes in the PB-020/anti-PD-1 combination versus control group. (**E**) Enrichment analysis of the KEGG pathway of the identified up-regulated genes in the PB-020/anti-PD-1 combination group versus control group. (**F**) Enrichment analysis of the KEGG pathway of the identified down-regulated genes in the PB-020/anti-PD-1 combination group versus control group. The size and color of the circles represent the number of enriched genes and the adjusted *p* value, respectively.

To unveil the molecular mechanism underlying this anticancer therapy, we executed RNA sequencing for the tumor samples obtained from the above experiments (Figure S8). The number of differentially expressed (DE) genes between the control (CK) group and PB-020 or anti-PD-1 treatment group, either singly or in combination, varied, with the combination group having the greatest number of DE genes (Figure 5A,B). GO cell compartment (CC) and GO molecular function (MF) analyses revealed that the DE genes in the PB-020/anti-PD-1 combination group versus the control group were significantly enriched in the “membrane” fraction and were associated with receptor and kinase binding (Figure S9). Subsequent GO biological process (BP) and KEGG pathway analyses revealed that the DE genes in the combination group versus the control group were mainly involved in the immune system, cell adhesion and PI3K/AKT pathway (Figure 5C,D), indicating a potential role of the PI3K/AKT pathway genes in mediating the therapeutic effects of the PB-020/anti-PD-1 combination.

Thus, we further examined the up- and down-regulated genes in the PB-020/anti-PD-1 combination group versus the control group, in the PB-020 group versus the control group and in the anti-PD-1 group versus the control group by KEGG analysis, respectively. It was evident that the PI3K/AKT pathway genes were down-regulated in all three compound treatment groups versus the control group, and the combination group versus the control group had the lowest *p* value (Figure 5E,F and Figure S10–S12), raising the possibility that PB-020 and anti-PD-1 may synergistically suppress the transcription of the PI3K/AKT pathway genes.

**Figure 6:**
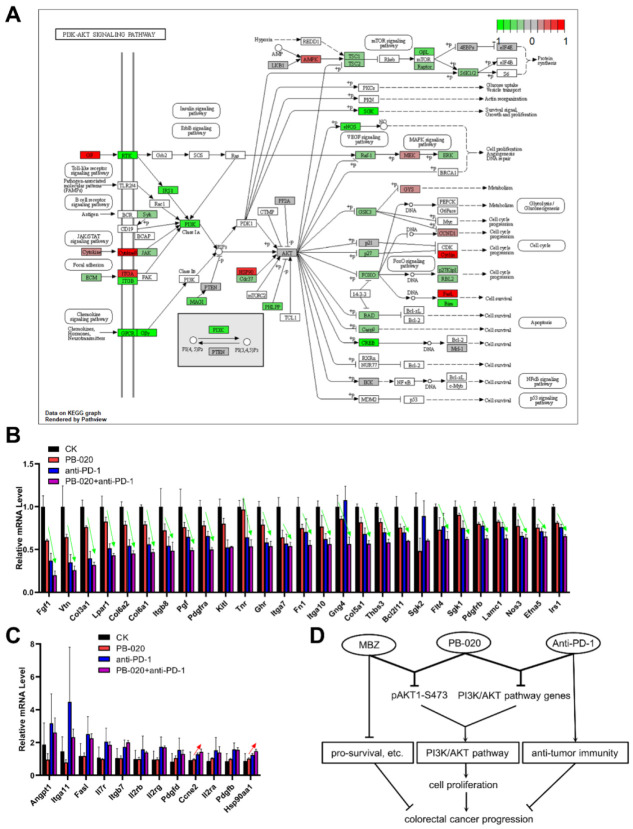
PB-020 in combination with PD-1 blockade suppressed the transcription of PI3K/AKT pathway genes. (**A**) The identified DE genes in the PB-020/anti-PD-1 combination group versus control group were largely involved in the PI3K/AKT signaling pathway. (**B**) Fold change in down-regulated PI3K/AKT pathway genes in the PB-020 group, anti-PD-1 group and PB-020/anti-PD-1 combination group versus the control, respectively. Green arrows indicate synergistic or additive suppression effects. (**C**) Fold change in up-regulated PI3K/AKT pathway genes in the PB-020 group, anti-PD-1 group and PB-020/anti-PD-1 combination group versus the control, respectively. Red arrows indicate synergistic or additive enhancement effects. (**D**) Graphical summary of the key findings of this work.

As shown in Figure 6A, multiple DE genes in the PB-020/anti-PD-1 combination group corresponded to key nodes of the PI3K/AKT signaling pathway, and most of them were down-regulated. To further nail down the most significantly changed genes by this combination treatment, we examined the expression fold changes and selected those PI3K/AKT-related DE genes whose transcription was the most significantly down- or up-regulated in the combination group. There were 27 genes that were found to be significantly down-regulated by the PB-020/anti-PD-1 combination treatment, and remarkably, 25 of them were synergistically or additively down-regulated (Figure 6B). In stark contrast, there were 12 genes that were found to be up-regulated by the combination treatment, but only 2 of them were synergistically or additively up-regulated (Figure 6C). These data strongly support the conclusion that PB-020 and anti-PD-1 can synergistically suppress the transcription of the PI3K/AKT pathway genes.

## 4. Discussion

Colorectal cancer (CRC) is generally diagnosed at an advanced stage, accompanied by tumor cell dissemination, when chemo- and targeted therapies can only achieve a limited increase in overall survival for these patients [24]. One of the key reasons for clinical failure lies in drug resistance, and the escape mechanisms of chemotherapy and targeted therapy are still the major cause of drug resistance [24]. Given that most cancers are essentially multifactorial diseases, the development of multitargeted therapies has grown rapidly, resulting in improved outcomes in various cancer models and selected multiple compound combinations that have been brought into clinical trials [25]. Consequently, combination therapy holds great promise in overcoming CRC drug resistance.

The activation of insulin/IGF-dependent pathways has been established as a key step that contributes to several mechanisms of CRC resistance to conventional and targeted therapeutic drugs [26], which makes it a promising target for CRC multi-target therapy. The human HCT 116 CRC cell line that overexpresses IGF-1R, instead of the parental cell line, was found to cause highly invasive tumors and induce distant metastases in murine models [27]. Moreover, IGF-1R overexpression was associated with AKT activation and up-regulation of the anti-apoptotic protein Bcl-xL [27]. In addition, IGF-1R was also found to participate in the activation of β-catenin in CRC cells [28]. Indeed, the knockdown of IGF-1R inhibited human CRC (HT-29 and SW620) cell growth by blocking the activation of downstream PI3K/AKT signaling, which, in turn, contributed to the activation of GSK3β. This protein inhibited β-catenin translocation into the nucleus and the transcription of cell proliferation genes [28]. Besides IGF-1R monotherapy, the combination of IGF-1R inhibitors with MEK1/2 inhibitors efficiently decreased the cell viability and increased apoptosis of CRC cells, overcoming the resistance to IGF-1R monotherapy [29]. Recently, we developed a novel PPP derivative (PB-020) that has better pharmacokinetic properties and higher BBB penetration capability than PPP [6]. Both PPP and PB-020 block IGF-1R signaling by competitively inhibiting the binding of IGF-1R to its native ligand IGF-1 [6]. The IGF-1 concentrations in culture media of mammalian cells [30], in culture media with supplemented IGF-1 [6], and in human serum [31] are 4 pM, 1.3 nM and 21.6 nM, respectively. Since the concentrations of PB-020 usually reach the μM range both in cell culture and in animal models [6], it is expected to be able to block IGF-1R signaling in those settings. However, the therapeutic efficacy of PB-020 against CRC remains untested thus far.

In this study, we tested the therapeutic efficacy of PB-020 in combination with the repurposed drug MBZ and the immunotherapeutic agent anti-PD-1 antibody against multiple in vitro and in vivo CRC models.

The anticancer activity of MBZ has been validated in multiple in vitro and in vivo studies. Although its mechanism of action remains unclear, MBZ is thought to be able to hit multiple drug targets. For instance, it can act as an anti-tubulin agent, inhibiting tubulin polymerization in several cancer cell lines, and is, therefore, applied as a substitute for vincristine when treating brain tumors [32]. Several preclinical studies have proven the efficacy of MBZ as an inhibitor against multiple processes that are accountable for tumor resistance and progression at nano- and micro-molar clinically reachable concentrations, including dysregulated angiogenesis, protein kinase activation and expression (including BCR–ABL, BRAF, VEGFR2 and MEK), matrix metalloproteinase 2 and multi-drug resistance protein transporters MRP1 and *p*-gp and pro-survival pathways (such as c-MYB, XIAP, SHH and MAPK/ERK) [9]. MBZ was also found to stimulate antitumoral immune responses by the polarization of macrophages towards M1 tumor-suppressive phenotypes in vitro [33]. In vivo, MBZ treatment, alone or with other anti-cancer compounds, exhibited reduced tumor growth and metastasis, suppressed angiogenesis, and prolonged survival [9]. Given its proved anticancer potency and desirable safety profile, MBZ could become a promising drug candidate to be combined with PB-020.

In recent years, great progress has been made in the treatment of various malignant tumors in the exploration of tumor immune checkpoints and their corresponding inhibitors [34]. PD-1, a transmembrane glycoprotein expressed in activated T cells, inhibits the activation and proliferation of T cells, and induces the immune escape of tumor cells when combined with its ligand PD-L1 [35]. PD-1/PD-L1 inhibitors block the signal transmission of PD-1/PD-L1, restoring the activity of T cells that can kill tumor cells to achieve therapeutic purposes. However, the efficacy of PD-1/PD-L1 inhibitors alone remains poor in the treatment of advanced CRC. PD-1/PD-L1 inhibitor monotherapy achieves good efficacy in dMMR/ MSI-H patients, but not pMMR/MSS patients [36]. Combination therapy is demonstrated to perform better than monotherapy in many cases. For example, the combination of PD-L1 inhibitors and MEK inhibitors could promote the efficacy of immunotherapy in MSS patients [37]. Furthermore, the combination of PD-1 inhibitors and HDAC inhibitors can produce synergistic effects against multiple cancers [38]. However, the current clinical results are not ideal for MSS patients, and more clinical trials and in-depth research are, therefore, needed to discover the optimal combination treatment regimen.

In this study, we demonstrated that the novel IGF1-R inhibitor PB-020 and the multi-target compound MBZ can synergistically reduce the viability of CRC cells and block xenograft CRC progress, which is accompanied by the synergistic down-regulation of the pAKT1-S473 level. Furthermore, PB-020 and anti-PD-1 antibodies can synergistically dampen CRC progression that is associated with the synergistic suppression of PI3K/AKT pathway genes (Figure 6D). Despite our observation of the potential synergism between PB-020 and MBZ, this combination warrants further investigation. Interestingly, both combinations appear to synergistically suppress the PI3K/AKT signaling pathway genes. The PB-020/MBZ combination potently reduced the level of activated pAKT1-S473 protein, as maximal activation of AKT requires phosphorylation of S473 in the hydrophobic motif in multiple solid tumors [39] and the potent inhibitory effect of PB-020 and MBZ on pAKT1-S473 holds therapeutic potential for treating CRC and other cancers. Interestingly, the PB-020/anti-PD-1 antibody combination synergistically suppressed the transcription of multiple PI3K/AKT pathway genes. These suppressed genes include IRS1, PDGFRA and PDGFRB. IRS1 and IRS2, the signaling components immediately downstream of the insulin/IR complex, are known to be critical mediators for CRC aggressiveness. Specifically, the IRS1 expression level appears to be inversely correlated to CRC differentiation, highlighting the role of IRS1 in CRC progression and liver metastasis, since IRS1 immunostaining is significantly higher in hepatic metastases relative to both primary CRC and paired colonic epithelium [40]. Likewise, elevated PDGFRα/β levels in the stroma of CRC patients were found to be correlated with metastasis [41] and recurrence [42]. PDGFRs are known to cross-talk with IGR-1R in many cancers [6,43], and PDGFRβ acts as a bypass resistance pathway to IGF-1R inhibition in rhabdomyosarcoma [44]. Therefore, the reduction in PDGFRs by the PB-020/anti-PD-1 combination may partially account for their synergistic therapeutic effect. Although the sequencing data were generated from xenografted tumors, a number of differential genes shown in Figure 6B appeared to be stromal in origin (such as Vtn and collagen), indicating a possible stromal impact on CRC that might involve ECM remodeling, fibroblast activation, and immune exclusion. Future in-depth investigations are warranted to fully understand the effects of the PB-020/anti-PD1 combination on changes in relative proportions, tumor infiltration and the antitumor activity of various immune cell types.

## 5. Conclusions

Taken together, in this study, a preclinical proof-of-concept for combating CRC has been established by combining multi-target treatment with PB-020 and clinical anti-tumor drugs.

## Data Availability

The data presented in this study are available upon request from the corresponding author. All sequencing data reported in this study have been submitted to the NCBI Gene Expression Omnibus database (https://www.ncbi.nlm.nih.gov/geo/, accessed on 20 August 2022) under accession number GSE189469.

## Supplementary Materials

The following supporting information can be downloaded at: https://www.mdpi.com/article/10.3390/cancers14235747/s1, Figure S1: Inhibitory effect of PB-020 and Taxol against HT-29 and SW480 colorectal cancer cells; Figure S2: Inhibitory effect of PB-020/FBZ or PB-020/MBZ against HT-29 colorectal cancer cells; Figure S3: Inhibitory effect of PB-020/FBZ or PB-020/MBZ against SW480 colorectal cancer cells; Figure S4: Inhibitory effect of PB-020 and MBZ against HCT 116 and LoVo colorectal cancer cells; Figure S5: Inhibitory effect of PB-020 and MBZ against SW620 colorectal cancer cells; Figure S6: Quantified band intensity in Figure S2; Figure S7: Data of tumor volumes in Figures S3C and S4C; Figure S8: Summary of RNA-seq reads analysis of tumor samples with the vehicle, PB-020, anti-PD-1 monoclonal antibody RMP1-14 or PB-020 + anti-PD-1 treatments; Figure S9: GO analysis of the identified differentially expressed (DE) genes in combined PB-020 + anti-PD-1 group versus control group; Figure S10: KEGG pathway analysis of up-regulated and down-regulated DE genes mentioned in Figure S4; Figure S11: GO analysis of DE genes in PB-020 group and anti-PD-1 group versus control group; Figure S12: KEGG pathway analysis of DE genes in PB-020 group and anti-PD-1 group versus control group.
