# Supplementary material for: The Novel IGF-1R Inhibitor PB-020 Acts Synergistically with Anti-PD-1 and Mebendazole against Colorectal Cancer
Source: Cancers (Basel). 2022 Nov 23;14(23):5747. doi: 10.3390/cancers14235747 (PMC9737525; doi:10.3390/cancers14235747)
Supplement: Supplementary file 1 [file cancers-14-05747-s001.zip › cancers-1908692-supplementary.pptx]

## Slide 1
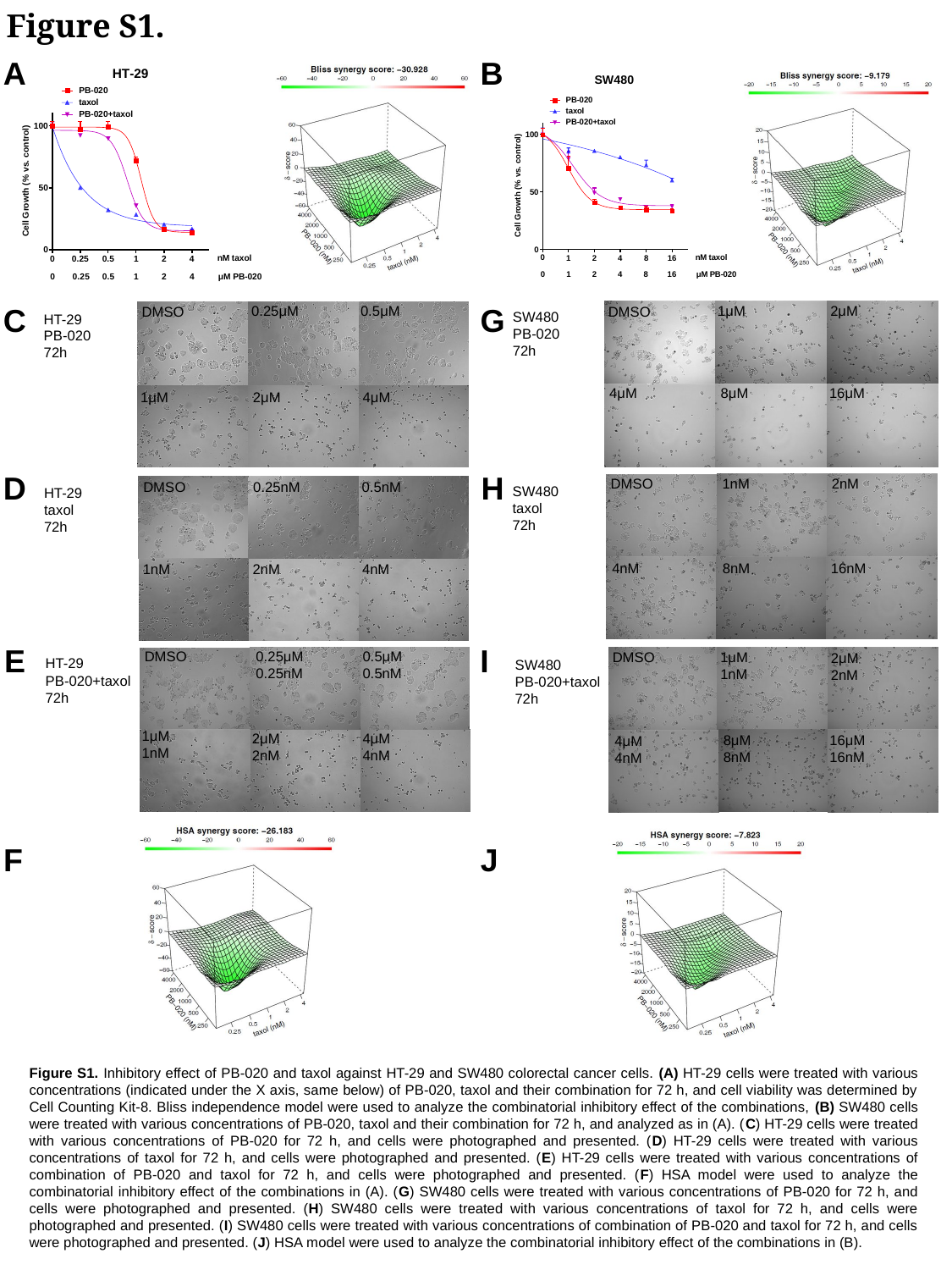

Figure S1.
A
B
1μM
2μM
C
G
0.25μM
0.5μM
DMSO
DMSO
SW480
PB-020
72h
HT-29
PB-020
72h
4μM
8μM
16μM
1μM
 2μM
4μM
D
H
DMSO
1nM
2nM
0.25nM
0.5nM
DMSO
SW480
taxol
72h
HT-29
taxol
72h
4nM
8nM
16nM
1nM
2nM
4nM
E
I
DMSO
0.25μM
0.25nM
0.5μM
0.5nM
1μM
1nM
DMSO
2μM
2nM
HT-29
PB-020+taxol
72h
SW480
PB-020+taxol
72h
1μM
1nM
2μM
2nM
4μM
4nM
16μM
16nM
8μM
8nM
4μM
4nM
F
J
Figure S1. Inhibitory effect of PB-020 and taxol against HT-29 and SW480 colorectal cancer cells. (A) HT-29 cells were treated with various concentrations (indicated under the X axis, same below) of PB-020, taxol and their combination for 72 h, and cell viability was determined by Cell Counting Kit-8. Bliss independence model were used to analyze the combinatorial inhibitory effect of the combinations, (B) SW480 cells were treated with various concentrations of PB-020, taxol and their combination for 72 h, and analyzed as in (A). (C) HT-29 cells were treated with various concentrations of PB-020 for 72 h, and cells were photographed and presented. (D) HT-29 cells were treated with various concentrations of taxol for 72 h, and cells were photographed and presented. (E) HT-29 cells were treated with various concentrations of combination of PB-020 and taxol for 72 h, and cells were photographed and presented. (F) HSA model were used to analyze the combinatorial inhibitory effect of the combinations in (A). (G) SW480 cells were treated with various concentrations of PB-020 for 72 h, and cells were photographed and presented. (H) SW480 cells were treated with various concentrations of taxol for 72 h, and cells were photographed and presented. (I) SW480 cells were treated with various concentrations of combination of PB-020 and taxol for 72 h, and cells were photographed and presented. (J) HSA model were used to analyze the combinatorial inhibitory effect of the combinations in (B).

## Slide 2
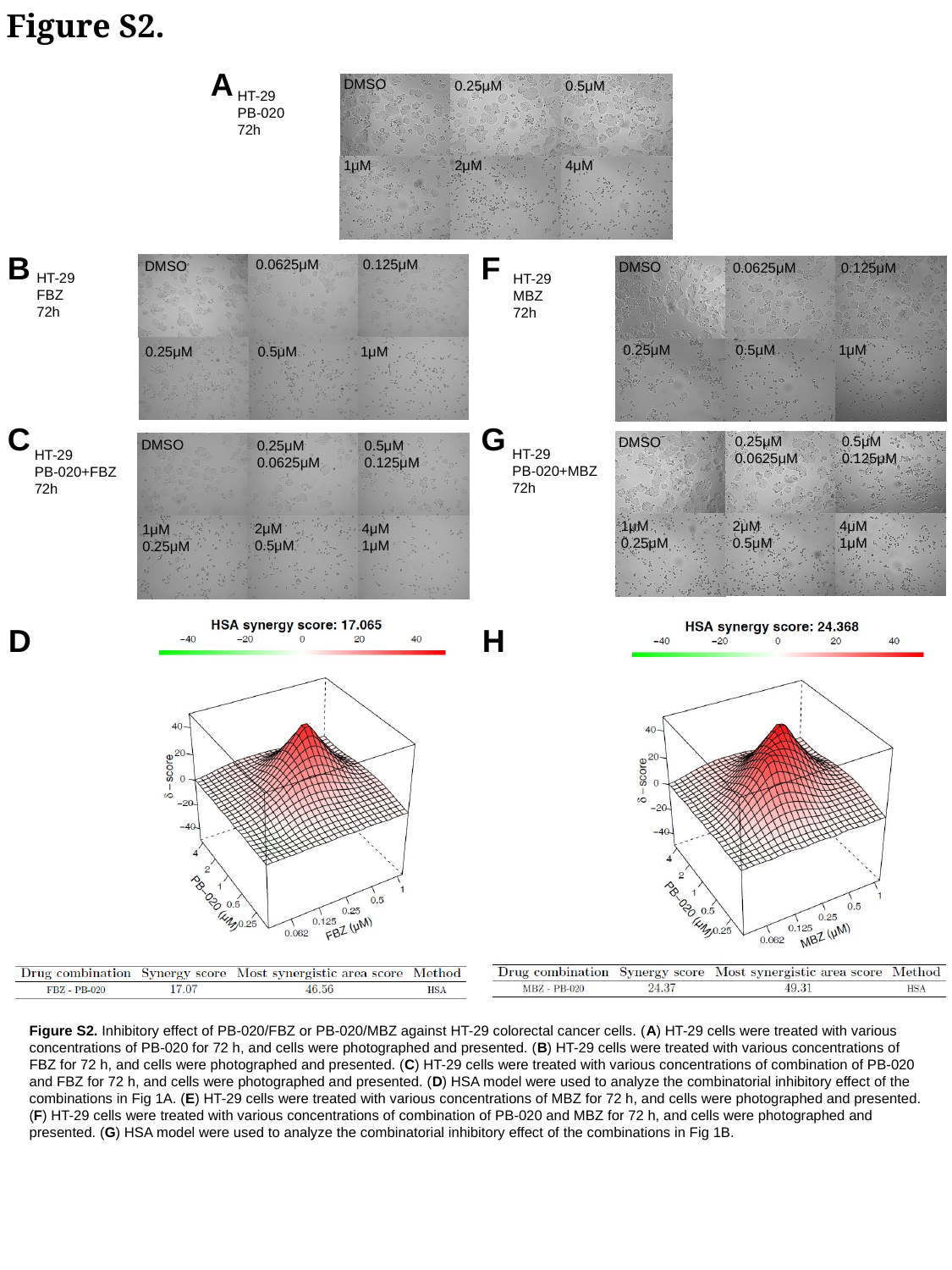

Figure S2.
A
DMSO
0.25μM
0.5μM
HT-29
PB-020
72h
2μM
4μM
1μM
B
F
0.0625μM
0.125μM
DMSO
DMSO
0.0625μM
0.125μM
HT-29
FBZ
72h
HT-29
MBZ
72h
0.25μM
 0.5μM
1μM
0.25μM
 0.5μM
1μM
C
G
0.25μM 0.0625μM
0.5μM 0.125μM
DMSO
DMSO
0.25μM 0.0625μM
0.5μM 0.125μM
HT-29
PB-020+MBZ
72h
HT-29
PB-020+FBZ
72h
1μM
0.25μM
2μM
0.5μM
4μM
1μM
2μM 0.5μM
4μM
1μM
1μM 0.25μM
D
H
Figure S2. Inhibitory effect of PB-020/FBZ or PB-020/MBZ against HT-29 colorectal cancer cells. (A) HT-29 cells were treated with various concentrations of PB-020 for 72 h, and cells were photographed and presented. (B) HT-29 cells were treated with various concentrations of FBZ for 72 h, and cells were photographed and presented. (C) HT-29 cells were treated with various concentrations of combination of PB-020 and FBZ for 72 h, and cells were photographed and presented. (D) HSA model were used to analyze the combinatorial inhibitory effect of the combinations in Fig 1A. (E) HT-29 cells were treated with various concentrations of MBZ for 72 h, and cells were photographed and presented. (F) HT-29 cells were treated with various concentrations of combination of PB-020 and MBZ for 72 h, and cells were photographed and presented. (G) HSA model were used to analyze the combinatorial inhibitory effect of the combinations in Fig 1B.

## Slide 3
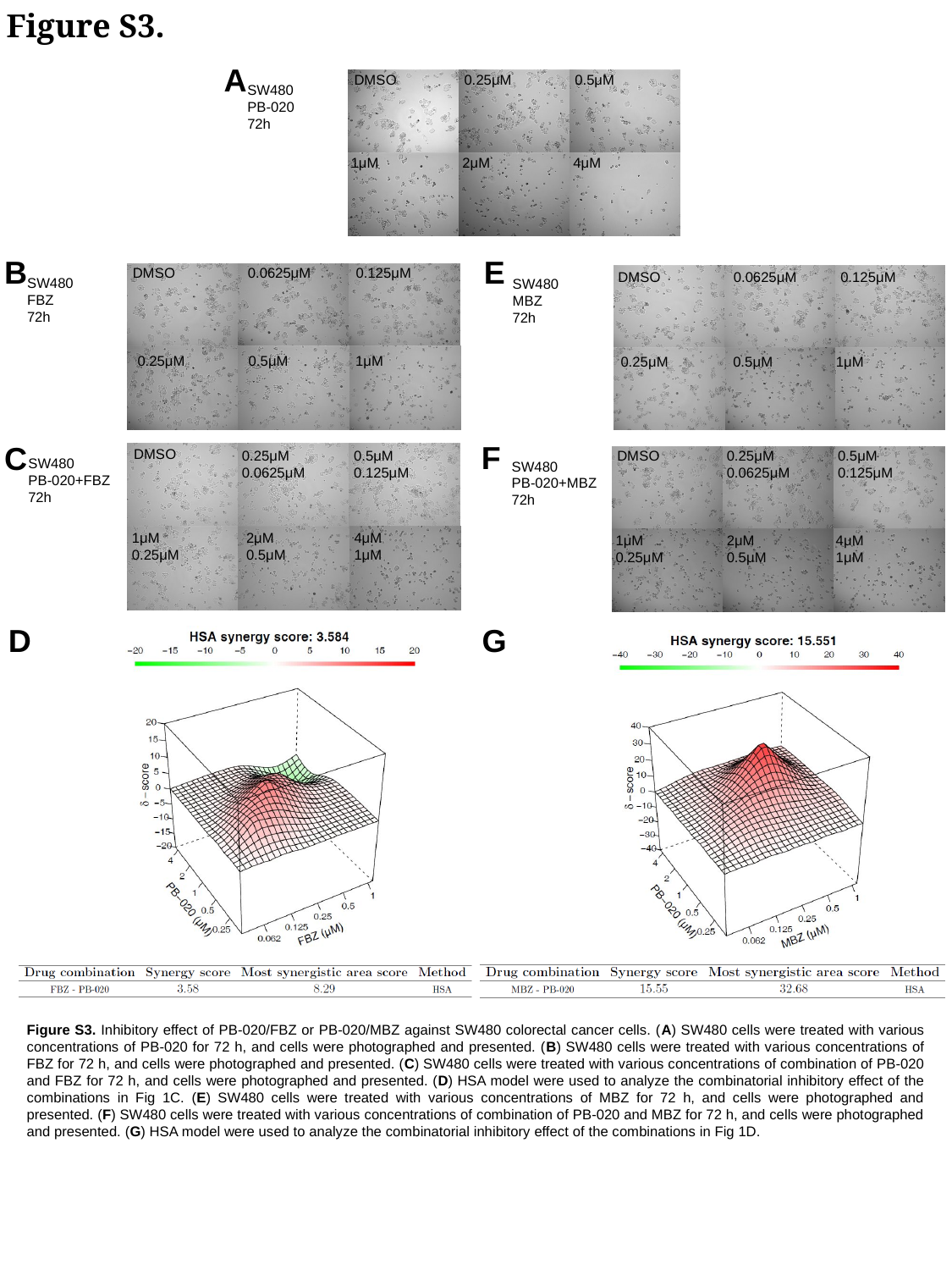

Figure S3.
A
DMSO
0.25μM
0.5μM
SW480
PB-020
72h
2μM
4μM
1μM
B
E
0.0625μM
0.125μM
DMSO
0.0625μM
0.125μM
DMSO
SW480
FBZ
72h
SW480
MBZ
72h
0.25μM
 0.5μM
1μM
0.25μM
 0.5μM
1μM
0.25μM
F
C
DMSO
0.25μM 0.0625μM
0.5μM 0.125μM
DMSO
0.25μM 0.0625μM
0.5μM 0.125μM
SW480
PB-020+FBZ
72h
SW480
PB-020+MBZ
72h
1μM 0.25μM
2μM 0.5μM
4μM
1μM
2μM 0.5μM
1μM
0.25μM
4μM
1μM
D
G
Figure S3. Inhibitory effect of PB-020/FBZ or PB-020/MBZ against SW480 colorectal cancer cells. (A) SW480 cells were treated with various concentrations of PB-020 for 72 h, and cells were photographed and presented. (B) SW480 cells were treated with various concentrations of FBZ for 72 h, and cells were photographed and presented. (C) SW480 cells were treated with various concentrations of combination of PB-020 and FBZ for 72 h, and cells were photographed and presented. (D) HSA model were used to analyze the combinatorial inhibitory effect of the combinations in Fig 1C. (E) SW480 cells were treated with various concentrations of MBZ for 72 h, and cells were photographed and presented. (F) SW480 cells were treated with various concentrations of combination of PB-020 and MBZ for 72 h, and cells were photographed and presented. (G) HSA model were used to analyze the combinatorial inhibitory effect of the combinations in Fig 1D.

## Slide 4
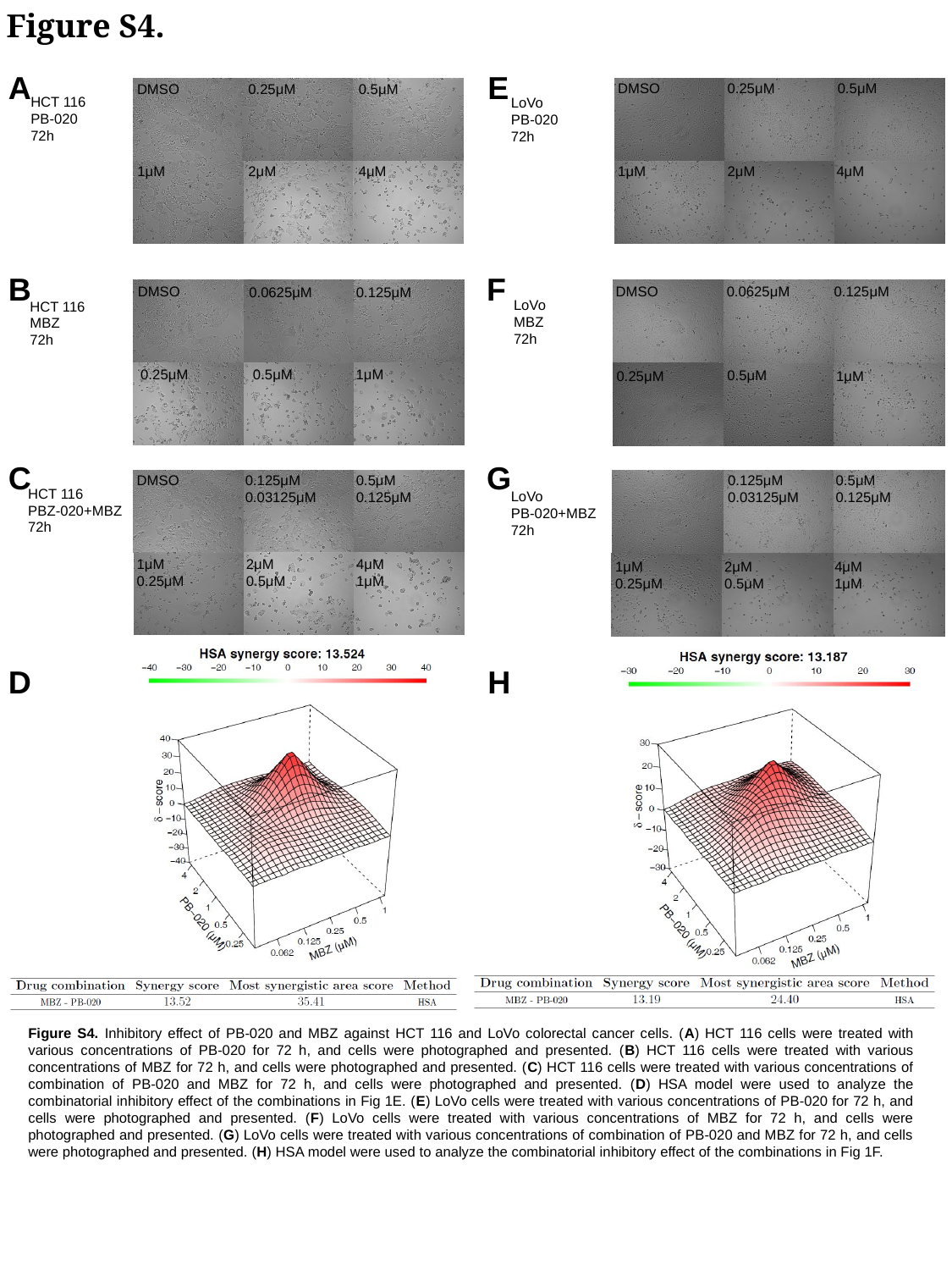

Figure S4.
A
E
DMSO
0.25μM
0.5μM
DMSO
0.25μM
0.5μM
HCT 116
PB-020
72h
LoVo
PB-020
72h
2μM
4μM
1μM
2μM
4μM
1μM
B
F
DMSO
0.0625μM
0.125μM
DMSO
0.0625μM
0.125μM
LoVo
MBZ
72h
HCT 116
MBZ
72h
0.25μM
 0.5μM
1μM
 0.5μM
0.25μM
1μM
C
G
DMSO
0.125μM 0.03125μM
0.5μM 0.125μM
DMSO
0.125μM 0.03125μM
0.5μM 0.125μM
HCT 116
PBZ-020+MBZ
72h
LoVo
PB-020+MBZ
72h
1μM
0.25μM
2μM
0.5μM
4μM
1μM
1μM
0.25μM
2μM
0.5μM
4μM
1μM
D
H
Figure S4. Inhibitory effect of PB-020 and MBZ against HCT 116 and LoVo colorectal cancer cells. (A) HCT 116 cells were treated with various concentrations of PB-020 for 72 h, and cells were photographed and presented. (B) HCT 116 cells were treated with various concentrations of MBZ for 72 h, and cells were photographed and presented. (C) HCT 116 cells were treated with various concentrations of combination of PB-020 and MBZ for 72 h, and cells were photographed and presented. (D) HSA model were used to analyze the combinatorial inhibitory effect of the combinations in Fig 1E. (E) LoVo cells were treated with various concentrations of PB-020 for 72 h, and cells were photographed and presented. (F) LoVo cells were treated with various concentrations of MBZ for 72 h, and cells were photographed and presented. (G) LoVo cells were treated with various concentrations of combination of PB-020 and MBZ for 72 h, and cells were photographed and presented. (H) HSA model were used to analyze the combinatorial inhibitory effect of the combinations in Fig 1F.

## Slide 5
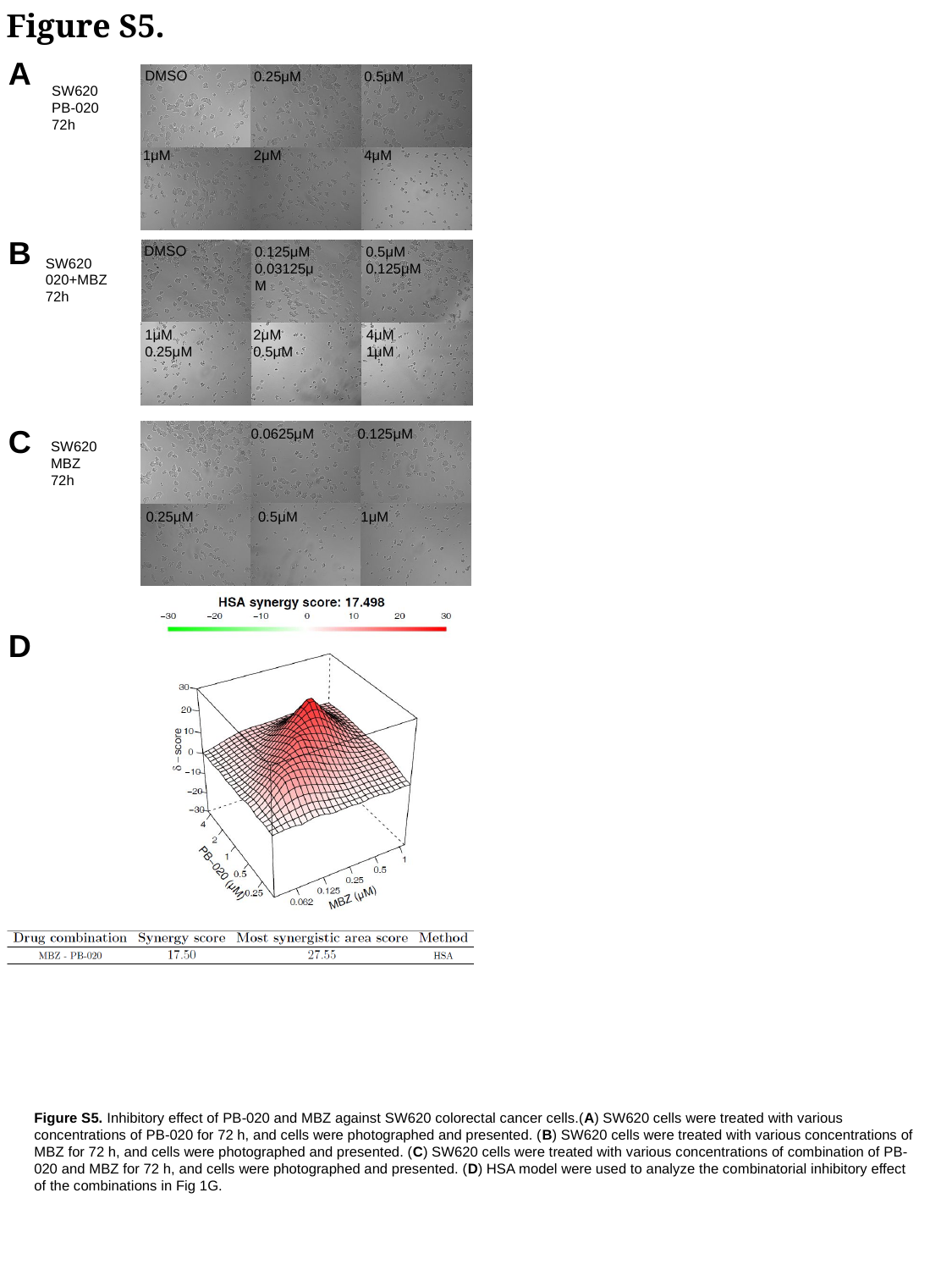

Figure S5.
A
DMSO
0.25μM
0.5μM
SW620
PB-020
72h
2μM
4μM
1μM
B
DMSO
0.125μM 0.03125μM
0.5μM 0.125μM
SW620
020+MBZ
72h
1μM
0.25μM
2μM
0.5μM
4μM
1μM
C
DMSO
0.0625μM
0.125μM
SW620
MBZ
72h
0.25μM
 0.5μM
1μM
D
Figure S5. Inhibitory effect of PB-020 and MBZ against SW620 colorectal cancer cells.(A) SW620 cells were treated with various concentrations of PB-020 for 72 h, and cells were photographed and presented. (B) SW620 cells were treated with various concentrations of MBZ for 72 h, and cells were photographed and presented. (C) SW620 cells were treated with various concentrations of combination of PB-020 and MBZ for 72 h, and cells were photographed and presented. (D) HSA model were used to analyze the combinatorial inhibitory effect of the combinations in Fig 1G.

## Slide 6
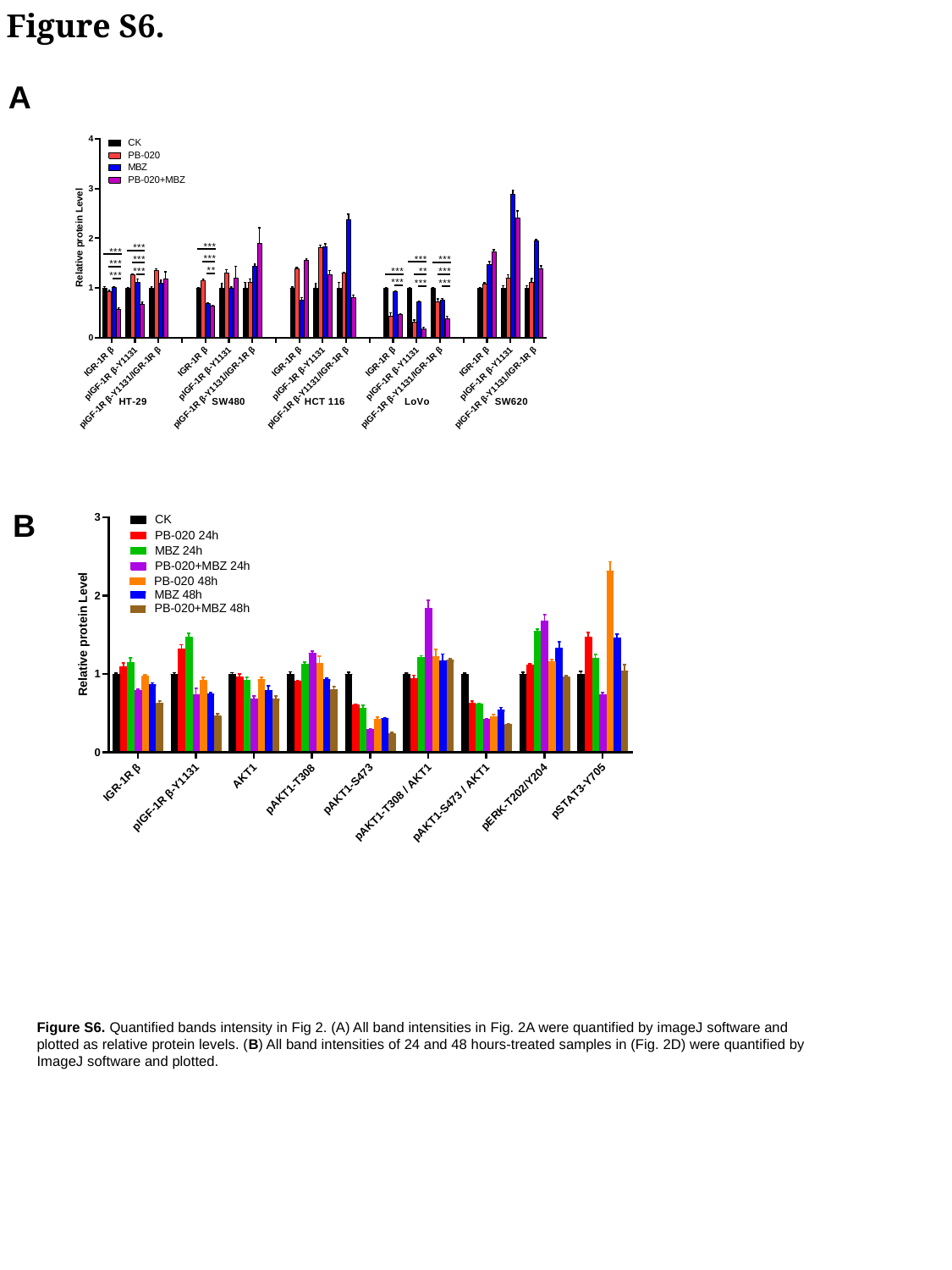

Figure S6.
A
B
Figure S6. Quantified bands intensity in Fig 2. (A) All band intensities in Fig. 2A were quantified by imageJ software and plotted as relative protein levels. (B) All band intensities of 24 and 48 hours-treated samples in (Fig. 2D) were quantified by ImageJ software and plotted.

## Slide 7
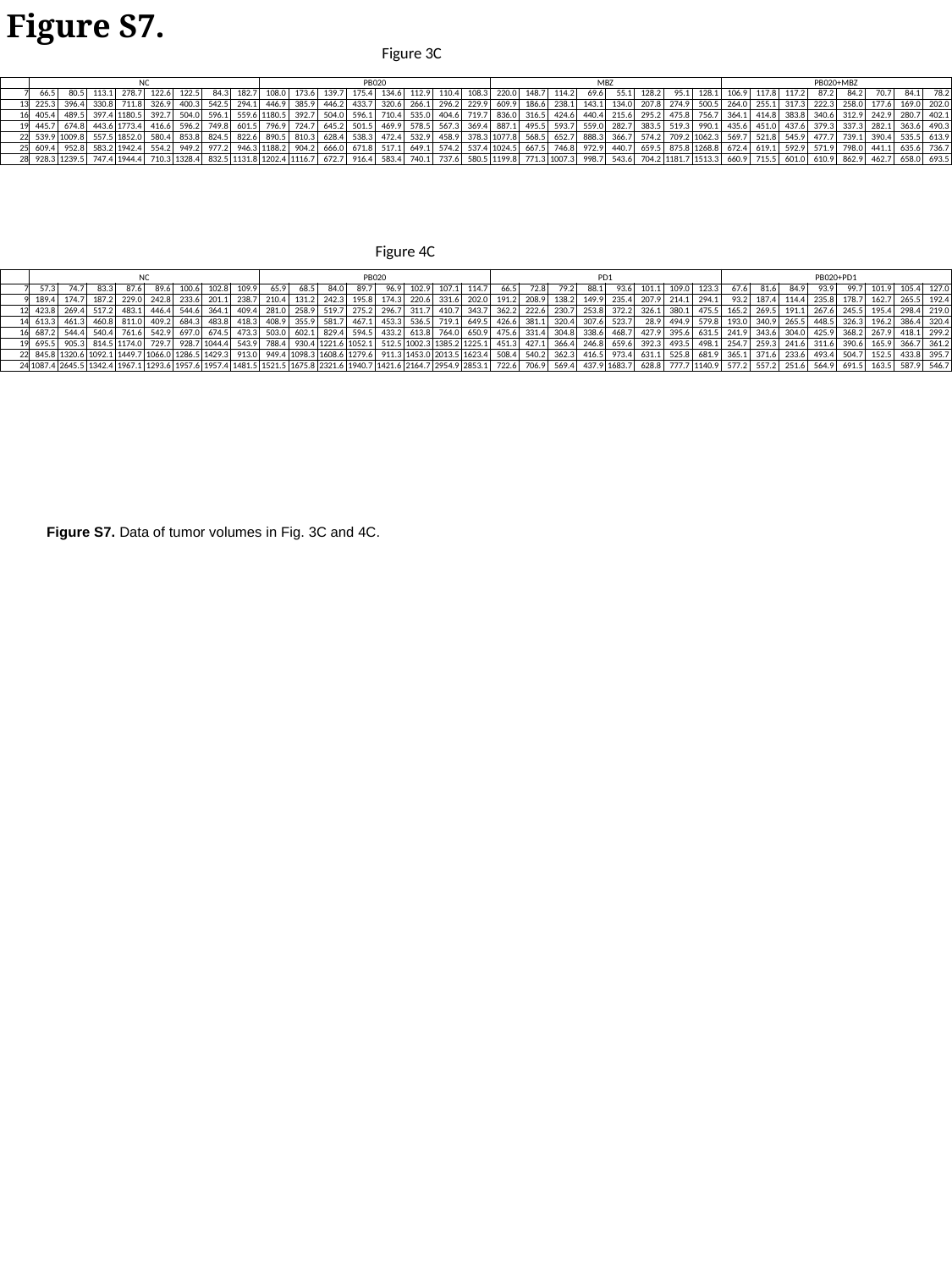

Figure S7.
Figure 3C
| | NC | | | | | | | | PB020 | | | | | | | | MBZ | | | | | | | | PB020+MBZ | | | | | | | |
| --- | --- | --- | --- | --- | --- | --- | --- | --- | --- | --- | --- | --- | --- | --- | --- | --- | --- | --- | --- | --- | --- | --- | --- | --- | --- | --- | --- | --- | --- | --- | --- | --- |
| 7 | 66.5 | 80.5 | 113.1 | 278.7 | 122.6 | 122.5 | 84.3 | 182.7 | 108.0 | 173.6 | 139.7 | 175.4 | 134.6 | 112.9 | 110.4 | 108.3 | 220.0 | 148.7 | 114.2 | 69.6 | 55.1 | 128.2 | 95.1 | 128.1 | 106.9 | 117.8 | 117.2 | 87.2 | 84.2 | 70.7 | 84.1 | 78.2 |
| 13 | 225.3 | 396.4 | 330.8 | 711.8 | 326.9 | 400.3 | 542.5 | 294.1 | 446.9 | 385.9 | 446.2 | 433.7 | 320.6 | 266.1 | 296.2 | 229.9 | 609.9 | 186.6 | 238.1 | 143.1 | 134.0 | 207.8 | 274.9 | 500.5 | 264.0 | 255.1 | 317.3 | 222.3 | 258.0 | 177.6 | 169.0 | 202.0 |
| 16 | 405.4 | 489.5 | 397.4 | 1180.5 | 392.7 | 504.0 | 596.1 | 559.6 | 1180.5 | 392.7 | 504.0 | 596.1 | 710.4 | 535.0 | 404.6 | 719.7 | 836.0 | 316.5 | 424.6 | 440.4 | 215.6 | 295.2 | 475.8 | 756.7 | 364.1 | 414.8 | 383.8 | 340.6 | 312.9 | 242.9 | 280.7 | 402.1 |
| 19 | 445.7 | 674.8 | 443.6 | 1773.4 | 416.6 | 596.2 | 749.8 | 601.5 | 796.9 | 724.7 | 645.2 | 501.5 | 469.9 | 578.5 | 567.3 | 369.4 | 887.1 | 495.5 | 593.7 | 559.0 | 282.7 | 383.5 | 519.3 | 990.1 | 435.6 | 451.0 | 437.6 | 379.3 | 337.3 | 282.1 | 363.6 | 490.3 |
| 22 | 539.9 | 1009.8 | 557.5 | 1852.0 | 580.4 | 853.8 | 824.5 | 822.6 | 890.5 | 810.3 | 628.4 | 538.3 | 472.4 | 532.9 | 458.9 | 378.3 | 1077.8 | 568.5 | 652.7 | 888.3 | 366.7 | 574.2 | 709.2 | 1062.3 | 569.7 | 521.8 | 545.9 | 477.7 | 739.1 | 390.4 | 535.5 | 613.9 |
| 25 | 609.4 | 952.8 | 583.2 | 1942.4 | 554.2 | 949.2 | 977.2 | 946.3 | 1188.2 | 904.2 | 666.0 | 671.8 | 517.1 | 649.1 | 574.2 | 537.4 | 1024.5 | 667.5 | 746.8 | 972.9 | 440.7 | 659.5 | 875.8 | 1268.8 | 672.4 | 619.1 | 592.9 | 571.9 | 798.0 | 441.1 | 635.6 | 736.7 |
| 28 | 928.3 | 1239.5 | 747.4 | 1944.4 | 710.3 | 1328.4 | 832.5 | 1131.8 | 1202.4 | 1116.7 | 672.7 | 916.4 | 583.4 | 740.1 | 737.6 | 580.5 | 1199.8 | 771.3 | 1007.3 | 998.7 | 543.6 | 704.2 | 1181.7 | 1513.3 | 660.9 | 715.5 | 601.0 | 610.9 | 862.9 | 462.7 | 658.0 | 693.5 |
Figure 4C
| | NC | | | | | | | | PB020 | | | | | | | | PD1 | | | | | | | | PB020+PD1 | | | | | | | |
| --- | --- | --- | --- | --- | --- | --- | --- | --- | --- | --- | --- | --- | --- | --- | --- | --- | --- | --- | --- | --- | --- | --- | --- | --- | --- | --- | --- | --- | --- | --- | --- | --- |
| 7 | 57.3 | 74.7 | 83.3 | 87.6 | 89.6 | 100.6 | 102.8 | 109.9 | 65.9 | 68.5 | 84.0 | 89.7 | 96.9 | 102.9 | 107.1 | 114.7 | 66.5 | 72.8 | 79.2 | 88.1 | 93.6 | 101.1 | 109.0 | 123.3 | 67.6 | 81.6 | 84.9 | 93.9 | 99.7 | 101.9 | 105.4 | 127.0 |
| 9 | 189.4 | 174.7 | 187.2 | 229.0 | 242.8 | 233.6 | 201.1 | 238.7 | 210.4 | 131.2 | 242.3 | 195.8 | 174.3 | 220.6 | 331.6 | 202.0 | 191.2 | 208.9 | 138.2 | 149.9 | 235.4 | 207.9 | 214.1 | 294.1 | 93.2 | 187.4 | 114.4 | 235.8 | 178.7 | 162.7 | 265.5 | 192.4 |
| 12 | 423.8 | 269.4 | 517.2 | 483.1 | 446.4 | 544.6 | 364.1 | 409.4 | 281.0 | 258.9 | 519.7 | 275.2 | 296.7 | 311.7 | 410.7 | 343.7 | 362.2 | 222.6 | 230.7 | 253.8 | 372.2 | 326.1 | 380.1 | 475.5 | 165.2 | 269.5 | 191.1 | 267.6 | 245.5 | 195.4 | 298.4 | 219.0 |
| 14 | 613.3 | 461.3 | 460.8 | 811.0 | 409.2 | 684.3 | 483.8 | 418.3 | 408.9 | 355.9 | 581.7 | 467.1 | 453.3 | 536.5 | 719.1 | 649.5 | 426.6 | 381.1 | 320.4 | 307.6 | 523.7 | 28.9 | 494.9 | 579.8 | 193.0 | 340.9 | 265.5 | 448.5 | 326.3 | 196.2 | 386.4 | 320.4 |
| 16 | 687.2 | 544.4 | 540.4 | 761.6 | 542.9 | 697.0 | 674.5 | 473.3 | 503.0 | 602.1 | 829.4 | 594.5 | 433.2 | 613.8 | 764.0 | 650.9 | 475.6 | 331.4 | 304.8 | 338.6 | 468.7 | 427.9 | 395.6 | 631.5 | 241.9 | 343.6 | 304.0 | 425.9 | 368.2 | 267.9 | 418.1 | 299.2 |
| 19 | 695.5 | 905.3 | 814.5 | 1174.0 | 729.7 | 928.7 | 1044.4 | 543.9 | 788.4 | 930.4 | 1221.6 | 1052.1 | 512.5 | 1002.3 | 1385.2 | 1225.1 | 451.3 | 427.1 | 366.4 | 246.8 | 659.6 | 392.3 | 493.5 | 498.1 | 254.7 | 259.3 | 241.6 | 311.6 | 390.6 | 165.9 | 366.7 | 361.2 |
| 22 | 845.8 | 1320.6 | 1092.1 | 1449.7 | 1066.0 | 1286.5 | 1429.3 | 913.0 | 949.4 | 1098.3 | 1608.6 | 1279.6 | 911.3 | 1453.0 | 2013.5 | 1623.4 | 508.4 | 540.2 | 362.3 | 416.5 | 973.4 | 631.1 | 525.8 | 681.9 | 365.1 | 371.6 | 233.6 | 493.4 | 504.7 | 152.5 | 433.8 | 395.7 |
| 24 | 1087.4 | 2645.5 | 1342.4 | 1967.1 | 1293.6 | 1957.6 | 1957.4 | 1481.5 | 1521.5 | 1675.8 | 2321.6 | 1940.7 | 1421.6 | 2164.7 | 2954.9 | 2853.1 | 722.6 | 706.9 | 569.4 | 437.9 | 1683.7 | 628.8 | 777.7 | 1140.9 | 577.2 | 557.2 | 251.6 | 564.9 | 691.5 | 163.5 | 587.9 | 546.7 |
Figure S7. Data of tumor volumes in Fig. 3C and 4C.

## Slide 8
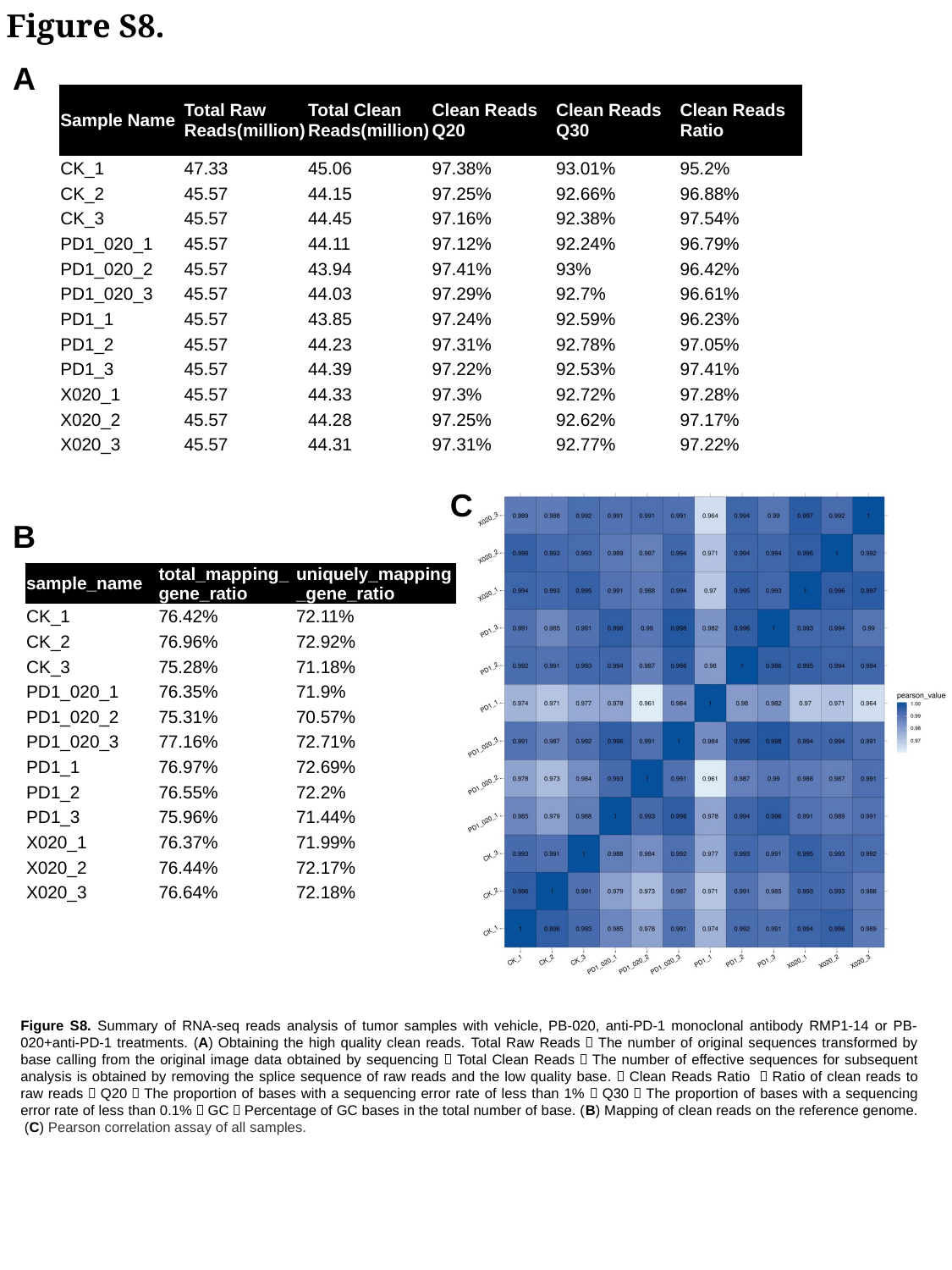

Figure S8.
A
| Sample Name | Total Raw Reads(million) | Total Clean Reads(million) | Clean Reads Q20 | Clean Reads Q30 | Clean Reads Ratio |
| --- | --- | --- | --- | --- | --- |
| CK\_1 | 47.33 | 45.06 | 97.38% | 93.01% | 95.2% |
| CK\_2 | 45.57 | 44.15 | 97.25% | 92.66% | 96.88% |
| CK\_3 | 45.57 | 44.45 | 97.16% | 92.38% | 97.54% |
| PD1\_020\_1 | 45.57 | 44.11 | 97.12% | 92.24% | 96.79% |
| PD1\_020\_2 | 45.57 | 43.94 | 97.41% | 93% | 96.42% |
| PD1\_020\_3 | 45.57 | 44.03 | 97.29% | 92.7% | 96.61% |
| PD1\_1 | 45.57 | 43.85 | 97.24% | 92.59% | 96.23% |
| PD1\_2 | 45.57 | 44.23 | 97.31% | 92.78% | 97.05% |
| PD1\_3 | 45.57 | 44.39 | 97.22% | 92.53% | 97.41% |
| X020\_1 | 45.57 | 44.33 | 97.3% | 92.72% | 97.28% |
| X020\_2 | 45.57 | 44.28 | 97.25% | 92.62% | 97.17% |
| X020\_3 | 45.57 | 44.31 | 97.31% | 92.77% | 97.22% |
C
B
| sample\_name | total\_mapping\_gene\_ratio | uniquely\_mapping\_gene\_ratio |
| --- | --- | --- |
| CK\_1 | 76.42% | 72.11% |
| CK\_2 | 76.96% | 72.92% |
| CK\_3 | 75.28% | 71.18% |
| PD1\_020\_1 | 76.35% | 71.9% |
| PD1\_020\_2 | 75.31% | 70.57% |
| PD1\_020\_3 | 77.16% | 72.71% |
| PD1\_1 | 76.97% | 72.69% |
| PD1\_2 | 76.55% | 72.2% |
| PD1\_3 | 75.96% | 71.44% |
| X020\_1 | 76.37% | 71.99% |
| X020\_2 | 76.44% | 72.17% |
| X020\_3 | 76.64% | 72.18% |
Figure S8. Summary of RNA-seq reads analysis of tumor samples with vehicle, PB-020, anti-PD-1 monoclonal antibody RMP1-14 or PB-020+anti-PD-1 treatments. (A) Obtaining the high quality clean reads. Total Raw Reads：The number of original sequences transformed by base calling from the original image data obtained by sequencing；Total Clean Reads：The number of effective sequences for subsequent analysis is obtained by removing the splice sequence of raw reads and the low quality base.；Clean Reads Ratio ：Ratio of clean reads to raw reads；Q20：The proportion of bases with a sequencing error rate of less than 1%；Q30：The proportion of bases with a sequencing error rate of less than 0.1%；GC：Percentage of GC bases in the total number of base. (B) Mapping of clean reads on the reference genome. (C) Pearson correlation assay of all samples.

## Slide 9
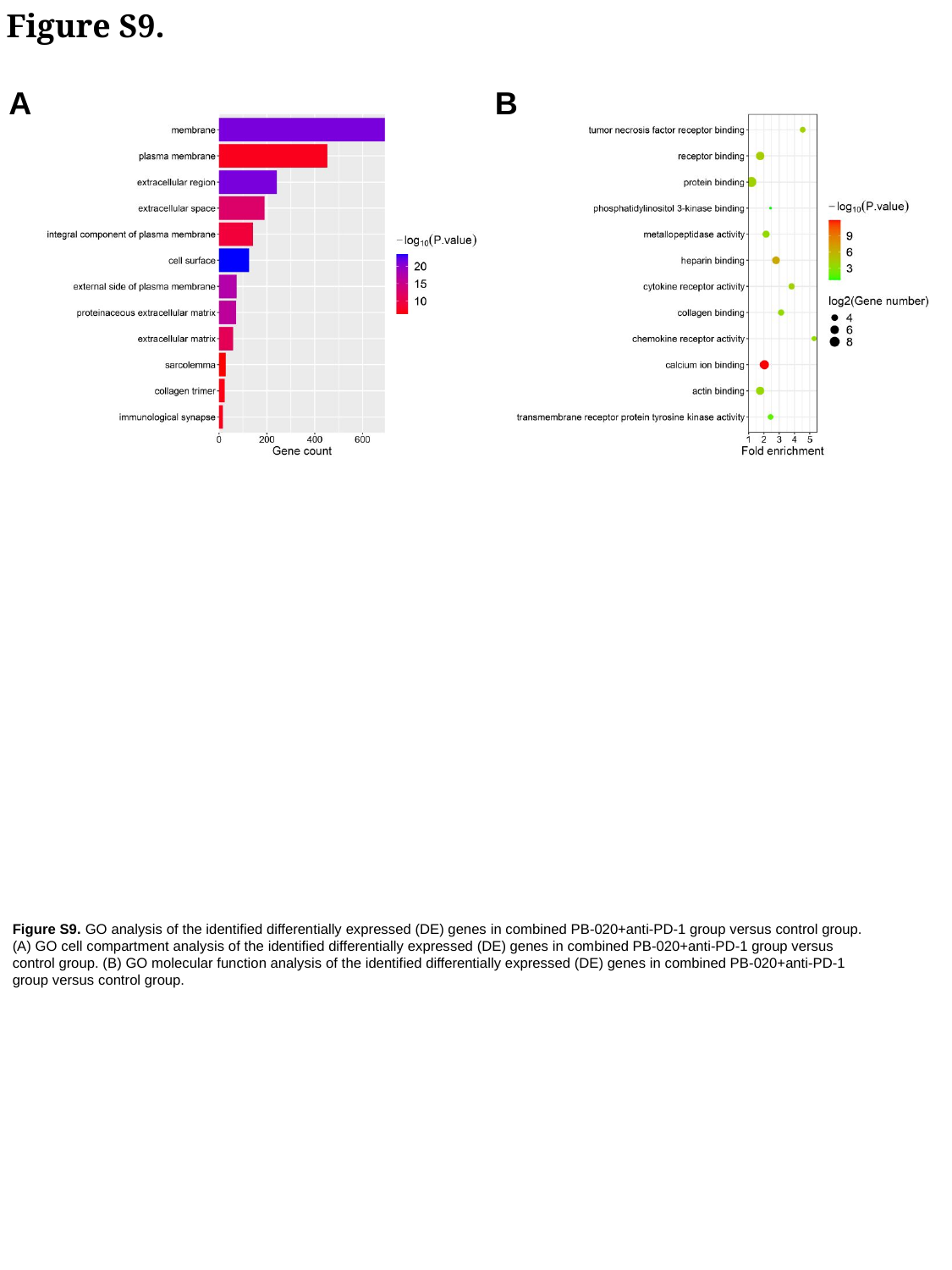

Figure S9.
A
B
Figure S9. GO analysis of the identified differentially expressed (DE) genes in combined PB-020+anti-PD-1 group versus control group. (A) GO cell compartment analysis of the identified differentially expressed (DE) genes in combined PB-020+anti-PD-1 group versus control group. (B) GO molecular function analysis of the identified differentially expressed (DE) genes in combined PB-020+anti-PD-1 group versus control group.

## Slide 10
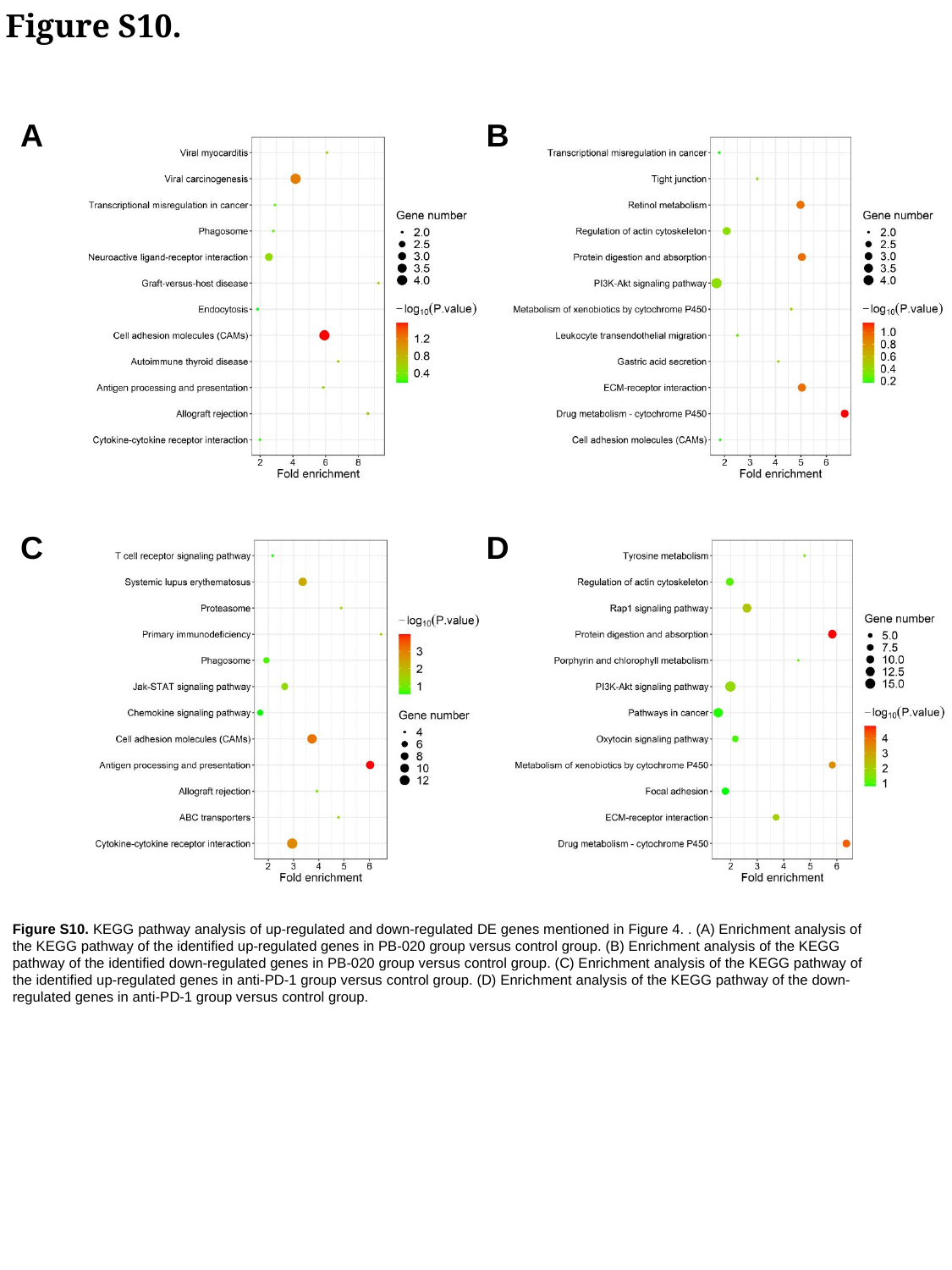

Figure S10.
A
B
C
D
Figure S10. KEGG pathway analysis of up-regulated and down-regulated DE genes mentioned in Figure 4. . (A) Enrichment analysis of the KEGG pathway of the identified up-regulated genes in PB-020 group versus control group. (B) Enrichment analysis of the KEGG pathway of the identified down-regulated genes in PB-020 group versus control group. (C) Enrichment analysis of the KEGG pathway of the identified up-regulated genes in anti-PD-1 group versus control group. (D) Enrichment analysis of the KEGG pathway of the down-regulated genes in anti-PD-1 group versus control group.

## Slide 11
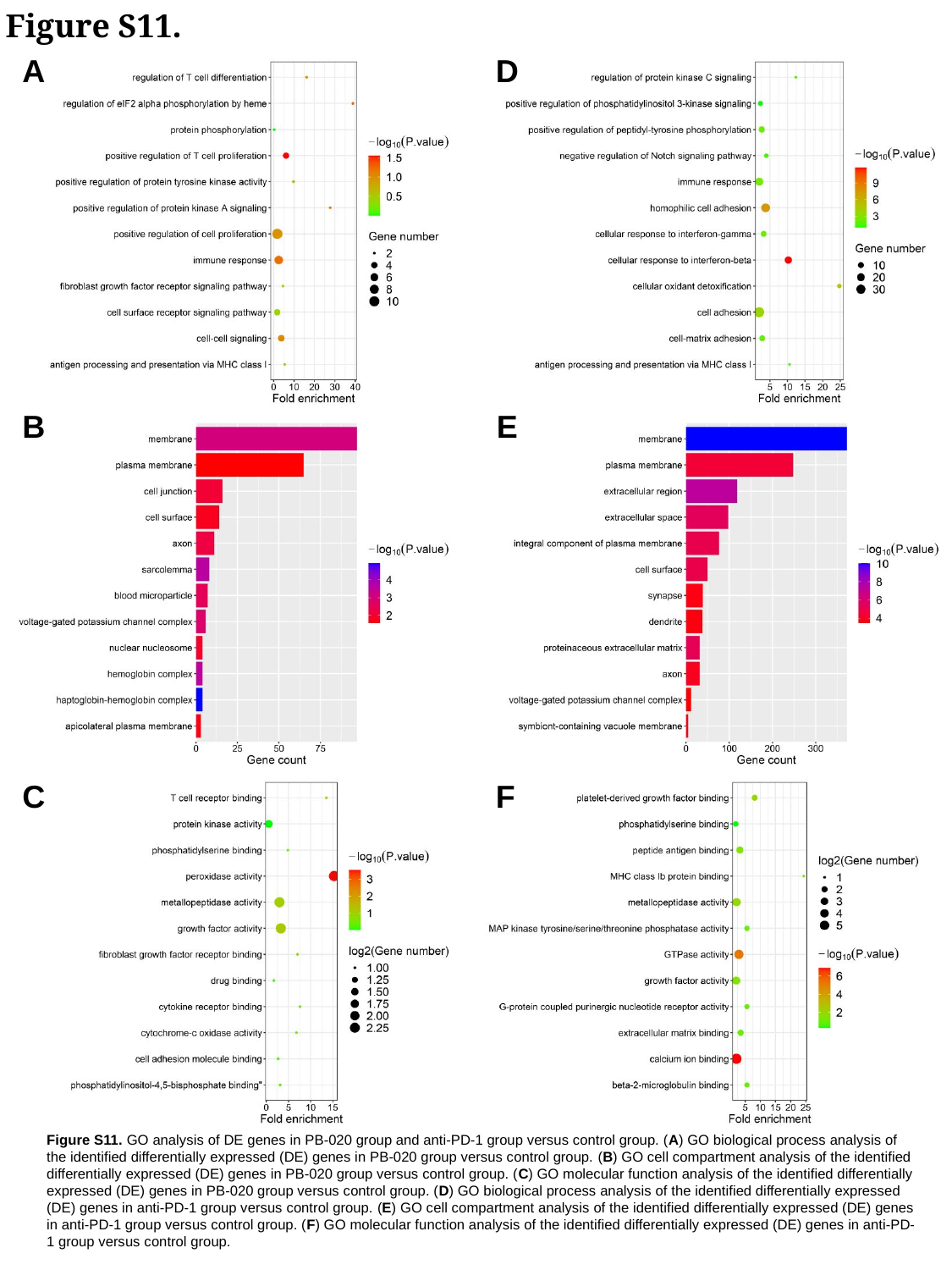

Figure S11.
A
D
B
E
C
F
Figure S11. GO analysis of DE genes in PB-020 group and anti-PD-1 group versus control group. (A) GO biological process analysis of the identified differentially expressed (DE) genes in PB-020 group versus control group. (B) GO cell compartment analysis of the identified differentially expressed (DE) genes in PB-020 group versus control group. (C) GO molecular function analysis of the identified differentially expressed (DE) genes in PB-020 group versus control group. (D) GO biological process analysis of the identified differentially expressed (DE) genes in anti-PD-1 group versus control group. (E) GO cell compartment analysis of the identified differentially expressed (DE) genes in anti-PD-1 group versus control group. (F) GO molecular function analysis of the identified differentially expressed (DE) genes in anti-PD-1 group versus control group.

## Slide 12
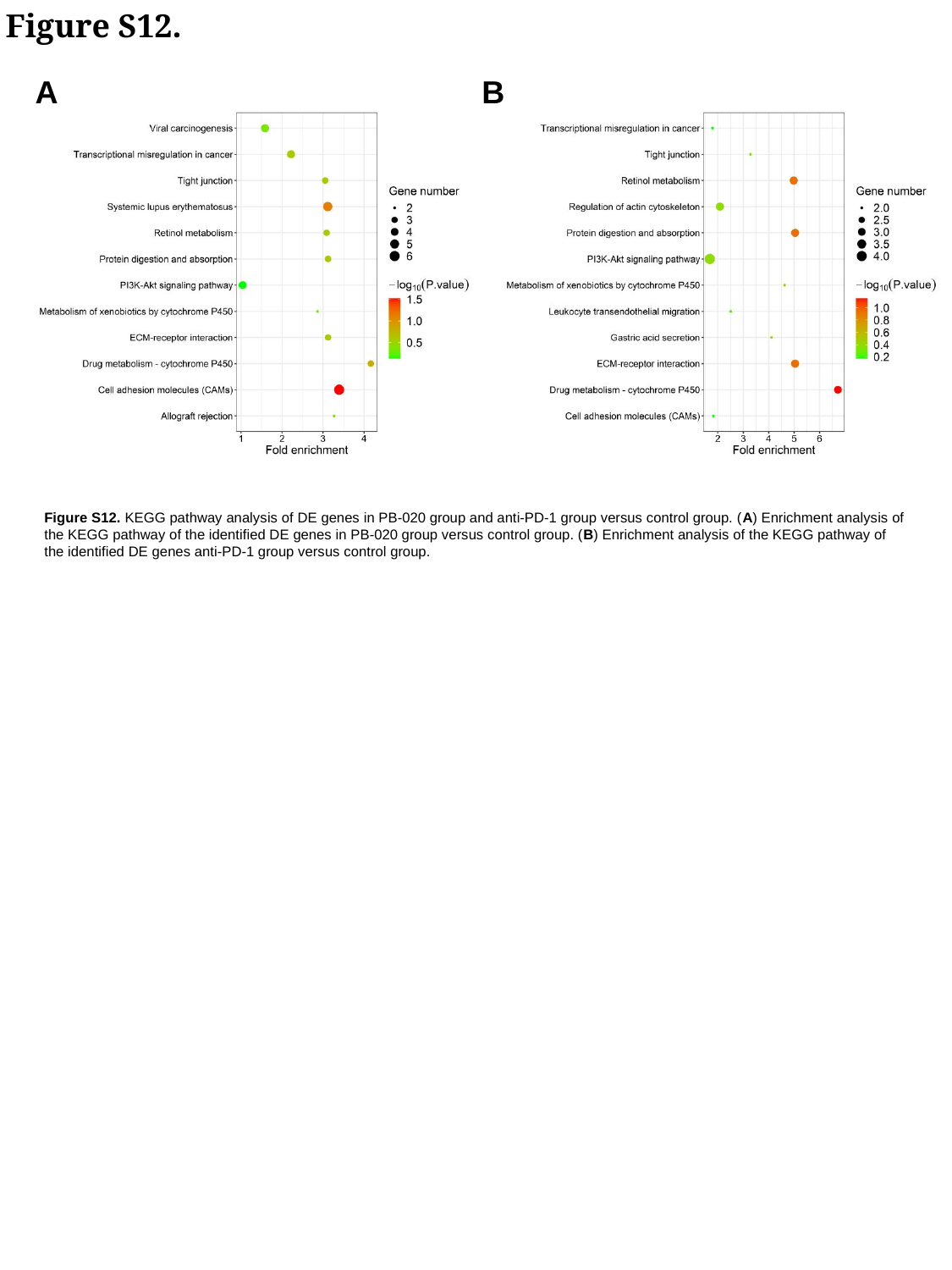

Figure S12.
A
B
Figure S12. KEGG pathway analysis of DE genes in PB-020 group and anti-PD-1 group versus control group. (A) Enrichment analysis of the KEGG pathway of the identified DE genes in PB-020 group versus control group. (B) Enrichment analysis of the KEGG pathway of the identified DE genes anti-PD-1 group versus control group.
